# Disentangling the impacts of collective mobility of residents and non-residents on burglary levels

**DOI:** 10.1007/s43762-025-00234-5

**Published:** 2026-01-13

**Authors:** Tongxin Chen, Kate Bowers, Tao Cheng

**Affiliations:** 1 Centre of Excellence for Data Science, Artificial Intelligence and Modelling, University of Hull, Cottingham Road, Hull, HU6 7RX UK; 2 Department of Security and Crime Science, University College London, Tavistock Square, London, WC1H 9EZ UK; 3 SpaceTimeLab for Big Data Analytics, University College London, Gower Street, London, WC1E 6BT UK

**Keywords:** Mobile phone GPS data, Human mobility, Explainable machine learning, Geo big data, Crime analysis

## Abstract

This study investigates how the collective mobility (including movement and visiting) of residents and non-residents affects neighbourhood burglary levels. While past research has linked mobility to urban crime, this study explores how these relationships vary across population groups and social contexts at the neighbourhood level. Using mobile phone GPS data, we distinguished between residents and non-residents based on daily movement patterns. We then measured their mobility within defined spatial and temporal units. An explainable machine learning method (XGBoost and SHAP) was used to assess how mobility patterns influence burglary in London’s LSOAs from 2020 to 2021. Results show that increased collective mobility is generally associated with higher burglary levels. Specifically, non-resident footfall and residents’ stay-at-home time have a stronger influence than other variables like residents’ travelled distance. The impact also varies across neighbourhoods and shifts during periods of COVID-19 restrictions and relaxations. These findings confirm the dynamic link between mobility and crime, highlighting the value of understanding population-specific patterns to inform more targeted policing strategies.

## Introduction

Collective human mobility refers to the aggregated patterns of individual or group movements and visits across geographic areas (Barbosa et al. 2018). Typically, human mobility is examined through two distinctive dimensions: movement, which captures flows and trajectories between locations and reflects the distances and extent people travel in daily life (Alessandretti et al. 2020; Gonzalez et al. 2008; Schläpfer et al. 2021), and visiting, which reflects frequency and duration at specific place/destinations, such as shopping centres or other social activity hubs (Papandrea et al. 2016; Chen et al. 2016). Understanding these mobility patterns is highly valuable for urban research as they reveal population movement and visiting across urban areas. Linking such collective mobility patterns to crime patterns can help to disentangle the spatio-temporal crime dynamics in urban neighbourhoods.

Opportunity theories suggest that crimes tend to concentrate in specific urban areas or locations characterised by high volumes of citizen activity and foot traffic providing opportunities for offenders to commit crimes (Brantingham and Brantingham 2016; Felson and Cohen 1980; Cohen and Felson 1979). This can be related to the population’s collective mobility patterns incorporating movement and visiting behaviours, which play a key role in the convergence of potential offenders, targets, and guardians within a specific urban space, thereby affecting crime opportunities (Levy et al. 2020; Cagney et al. 2020). For example, a bustling commercial street with high footfall traffic during business hours might experience higher theft crime rates due to the increased opportunities to attract offenders. Conversely, residential areas with little resident activity during working hours might be more susceptible to burglaries due to a lack of guardianship (Browning et al. 2010).

In addition, social disorganisation theories argue that crime is not simply the result of individual factors but is also influenced by the socio-economic characteristics of the geographical areas (e.g., neighbourhoods) where residents live. This phenomenon is commonly called the neighbourhood effect, which refers to the fact that crime rates tend to be higher in disadvantaged neighbourhoods whose residents tend to have difficulty in developing social cohesion and informal social control against crime occurrences (Shaw and McKay 1942; Sampson and Groves 1989; Sampson et al. 1997; Graif et al. 2014). While static socioeconomic factors can strongly impact the crime rates in neighbourhoods, exploring human mobility can further explain the movement and interactions of both residents and outsiders that influence crime levels. Previous studies have examined the connections between crime levels and neighbourhood disadvantage measured by the residents’ mobility dynamics conditions in neighbourhoods (Levy et al. 2020; Browning et al. 2020; White and Renk 2012).

The nexus between opportunity and neighbourhood theoretical approaches suggests a complex interplay between localised crime opportunities and broader neighbourhood contextual characteristics, leading to a mixed-effect understanding of crime patterns in urban neighbourhoods. Simply put, crime rates tend to be higher in urban areas with low social cohesion and informal social control (i.e., more disadvantaged neighbourhoods), and with high crime opportunities as well as low levels of guardianship (Sampson and Groves 2017; Cohen and Felson 1979).

Previous crime studies have used census data to evaluate social disorganisation in disadvantaged neighbourhoods, specifically considering factors such as poverty, unemployment, residential instability and deprivation (Sampson and Groves 1989; Sampson and Raudenbush 1999; Kawachi et al. 1999; De Courson and Nettle 2021; Boggess and Hipp 2010). In addition, static geographic land use or specific types of place data have been employed to measure crime (opportunity) generator levels, especially representing the areas frequently visited by a significant portion of the population (Brantingham and Brantingham 1995; Eck and Weisburd 2015; Kinney et al. 2008). In recent years, there has been a growing interest in utilising geo big data for sensing the collective mobility of populations as an alternative to traditional static data in the interpretation of crime patterns. This emerging stream also focuses on discussions regarding the population’s impact on creating opportunities or offering protection and contribution to the social conditions of neighbourhoods (Sampson and Levy 2020; Jones and Pridemore 2019; De Nadai et al. 2020). An initial aspect of interest is that geo big data can identify spatial and temporal collective activity patterns of populations, such as the number of people present in a specific area, including those who work, live, or visit there at a given time. This has enabled the evaluation of how collective mobility dynamically influences crime opportunities in urban areas.

A common approach involves examining geo big data gathered from location-based services or mobile service towers shared by numerous users to estimate the population collective mobility (movement and visiting) and link it with crime patterns in urban regions, including geo-tagged social media data (Malleson and Andresen 2015) and Call Detail Records (CDR) data (Bogomolov et al. 2014; Long et al. 2021; De Nadai et al. 2020; He et al. 2020; Zhang et al. 2022; Rumi et al. 2018; Tai et al. 2022). Notably, some studies have also leveraged sensed population *activities* (i.e., footfalls in urban areas) instead of arbitrarily quantifying the presence of ambient population using the numbers of users in datasets (Chen et al. 2023, 2022). This is because measuring visits or stays to represent population activity is more directly related to individual exposure to others, and thus better reflects the population’s activities across urban regions (Chen et al. 2022).

The second aspect of interest in geo big data and crime analysis has concentrated on utilising the flow data format (information for the origin and destination of population movements) to assess the commuting or travel patterns of the population linked to crime opportunities across urban regions, such as crowd-sourced mobile phone user’s movement flow (Wu et al. 2022; Kadar et al. 2020), transportation flows or trips (Kadar and Pletikosa 2018; Song et al. 2018, 2019). Additionally, in assessing the relationship between resident mobility and crime, unlike some earlier studies that relied on census population flow data (Graif et al. 2017, 2021; Browning et al. 2017), recent research has begun to explore more precise measures of resident collective mobility patterns to better understand crime dynamics across neighbourhoods. For example, Levy et al. (2020) used Twitter social media data sets to measure residents’ collective mobility and how it affects crime levels, and indicated that trends in structural mobility (flows) obtain a notable impact on neighbourhood homicides. Other studies have revealed that residents’ mobility patterns (e.g., residents frequently moving in and out of a neighbourhood) can influence levels of neighbourhood disorganisation, consequently disrupting the establishment of community cohesion which can help prevent crimes (Browning et al. 2017, 2020).

While empirical studies have linked population mobility to crime levels either through the lens of opportunity or neighbourhood theories, there has been no extensive investigation into the specific influence of different *types* of collective mobility from non-residents or residents on crime in neighbourhoods. The availability of high-resolution geo big data can facilitate the exploration of such different dimensions of sensed collective mobility patterns in space and time. This offers a valuable opportunity to explore how crimes are impacted by collective mobility measured in a way that can separate the movements of residents and visitors in neighbourhoods. In addition, human mobility experienced substantial changes during the COVID-19 pandemic due to social distancing measures in global cities (Hu et al. 2021; Galeazzi et al. 2021; Cheng et al. 2022). These changes affected both the population movement and visiting behaviours, which also alter opportunities for crime across urban neighbourhoods. Studying collective mobility during this period is therefore very useful in understanding how disruptions in normal social conditions influence crime dynamics. Analysing these variations enables us to understand the context-dependent mechanisms through which mobility behaviour influences crime and offers insights for developing adaptive prevention strategies under extraordinary societal conditions.

Thus, this study focuses on comprehensively integrating opportunity and neighbourhood theories when examining the influence of collective mobility on urban crime, particularly distinguishing the effects of residents’ activities from those of non-residents in neighbourhoods. In this study, we focus on the two-dimensional aspects of movement and visiting because they capture complementary aspects of collective mobility that are directly relevant to crime patterns (Browning et al. 2017, 2020). By disentangling these two dimensions, we are better able to evaluate how mobility behaviours of residents and non-residents differentially influence burglary risk in urban neighbourhoods. Burglary was selected as the crime type for this research as prior studies have shown that the patterns and prevalence of burglary are closely linked to a variety of identified factors, including residents’ activities and the overall social cohesion within the neighbourhood community (Bernasco and Luykx 2003; Nobles et al. 2016). Neighbourhoods with more resident interaction and stronger community connections typically have lower rates of burglary. In contrast, areas where social engagement is limited and community bonds are weaker tend to see higher rates of burglary (Cancino 2003; Markowitz et al. 2001; Sampson and Groves 2017). This correlation highlights the suitability of using burglary as a case study when considering collective mobility in analysing crime patterns within neighbourhoods. Burglary has also been shown to be sensitive to shifting contexts, and studies have shown a relationship between burglary patterns and the mobility restrictions that were enforced during the COVID pandemic (Halford et al. 2020; Mohler et al. 2020).

In summary, the research questions for the current study are: (1) How does the collective mobility of residents and non-residents affect crime occurrences in local neighbourhoods? (2) Does this impact differ across neighbourhoods with different conditions (e.g., different levels of social disorganisation)? (3) Additionally, does this impact change during different societal conditions, such as during the pandemic?

This paper is organised as follows: The Data and methods section introduces the data used in the study area and explains how this research defined collective mobility patterns (both movement and visiting) for separate resident and non-resident populations. It also describes the explainable machine learning techniques (i.e., XGBoost and SHAP) employed in this study. The result section presents and compares both global and local interpretations of the impact of mobility on burglary across a number of different modelling exercises across the study area of London during a two-year observation period covering the pandemic. The discussion section explores the significance and insights drawn from the results and considers the limitations. Finally, the conclusions section summarises the key findings and potential future works from this research.

## Data and methods

### Data and study area

As the case study for this analysis, London had a population of over 9.6 million in 2023. The metropolis comprises 33 local authorities (LAs) and 4,835 local neighbourhood areas officially referred to as Lower Super Output Areas (LSOAs) in the UK census. For this study, the LSOA geographical boundaries (as the primary geographical unit of analysis) in London were obtained from the ONS data protocol^1^. The areas of London LSOAs ranged from 18,362 $m^2$ to 15,797,244 $m^2$, with a mean of 329,828 $m^2$ and a standard deviation of 638,819 $m^2$. In the UK context, the LSOA is commonly adopted as the unit of analysis to reduce analytic complexity in crime studies (Malleson and Andresen 2015; Tompson et al. 2015).

Burglary incident data covering the period from 2020 to 2021 were downloaded from the Metropolitan Police Service section of the UK Online Police Data Portal^2^. The data include each crime event with corresponding spatial (latitude, longitude, LSOA index) and temporal (month and year) information.

The mobile phone GPS trajectory data set, including spatial coordinates and timestamps for each point, was anonymously collected from various applications (e.g., navigation, route planning, and outdoor sports) that utilise location-based services (LBS)^3^. This anonymous mobile phone data collection was conducted in compliance with user agreements established under the General Data Protection Regulation (GDPR) to guarantee the privacy and security of user information. This study included 1,979,081 users representing approximately 22% of the total resident population in London during the two-year observation period from 2020 to 2021.

As the key neighbourhood condition factor considered in understanding crime patterns (Bursik Jr and Grasmick 1993; Messer et al. 2006), the latest urban deprivation indices (2019 version) data in London were downloaded from the Ministry of Housing, Communities & Local Government website^4^. The ‘Indices of Deprivation’ data of London is the measurement of several types of deprivation for the 4,835 LSOAs. The main index used in this work is called the ‘Index of Multiple Deprivation (IMD)’, which combines weighted measurement across seven distinct subtypes of aspects of deprivation, including ‘Income Score (rate)’, ‘Employment Score (rate)’, ‘Education, Skills and Training Score’, ‘Health Deprivation and Disability Score’, ‘Crime Score’, ‘Barriers to Housing and Services Score’ and ‘Living Environment Score’. As the IMD measures the level of deprivation within a neighbourhood reflecting the overall socio-economic conditions of the area, it is a commonly used variable in crime studies across the UK (Lymperopoulou and Bannister 2022; Weir 2019).

### Characterising collective mobility (movement and visiting) from mobile phone GPS data

**Fig. 1:**
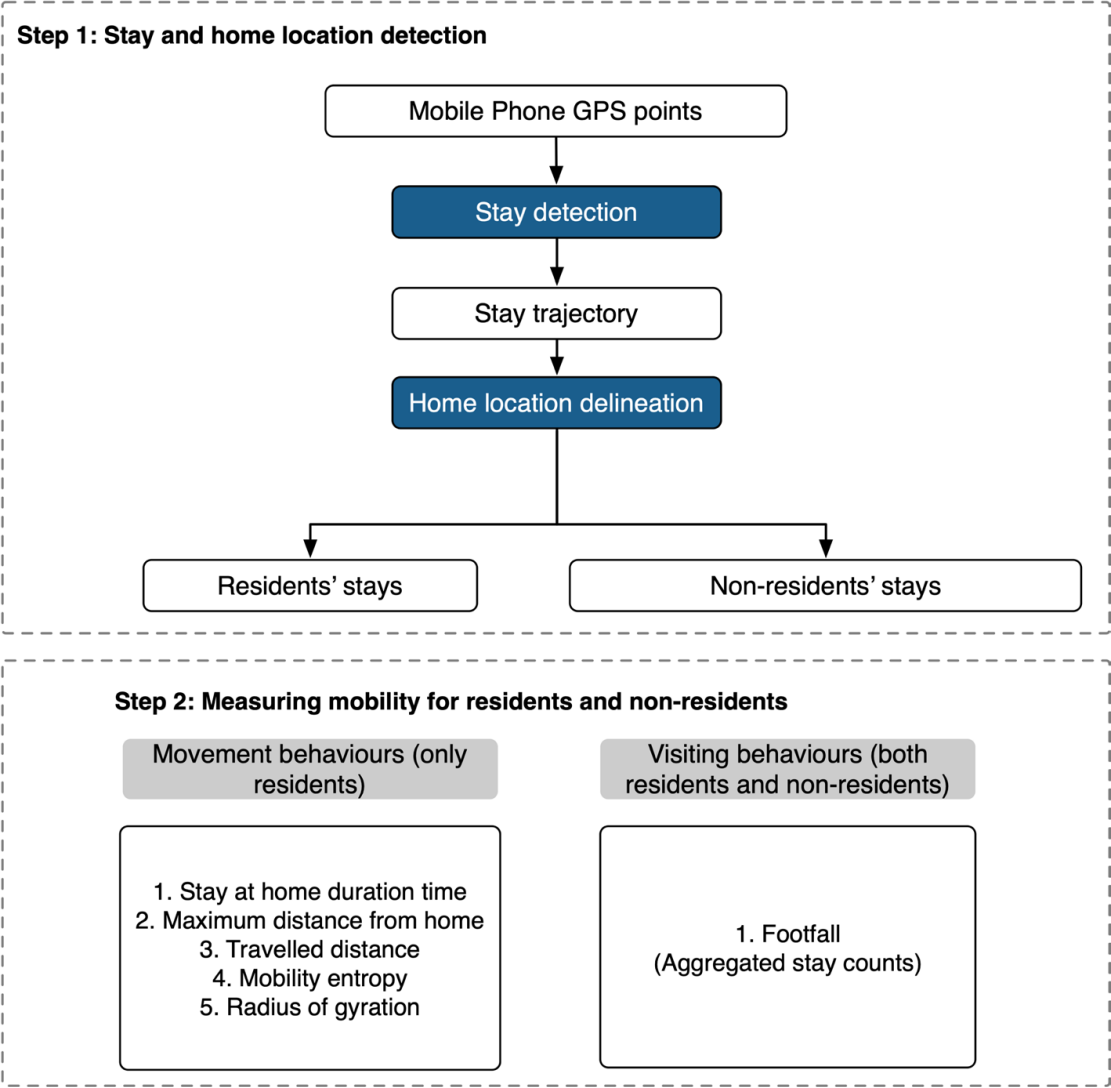
The measurement of movement and visiting behaviours for residents and non-residents based on GPS mobile phone trajectory data

To characterise collective mobility (movement and visiting behaviours) within the study’s spatio-temporal units (i.e., the LSOA-level and month-level) of different groups of populations, the GPS data obtained from anonymous users were prepared and analysed. The detection and measurement of movement and visiting behaviours for residents and non-residents are presented in Fig. 1 and consist of two main steps: The objective of ‘Step 1’ is to differentiate between residents and non-residents by identifying users’ stay patterns and then determining the home location based on daily GPS trajectory data from users’ mobile phones. Then, for each observation spatial and temporal unit/grid, ‘Step 2’ defines and measures the *movement behaviours* for residents only, including stay-at-home duration time, maximum distance from home, travelled distance, mobility entropy and radius of gyration. A separate variable is then created in ‘Step 2’ which represents *visiting behaviours*, i.e., aggregated stays as footfalls that are not at home or work locations, for both resident and non-resident groups.

To clarify the mobility behaviour variables for residents and non-residents, Table 1 outlines the main definitions/descriptions of residents, non-residents and collective mobility variables used in this study.

**Table 1:** A checklist for the definitions/descriptions of residents, non-residents and mobility variables used in this study

Name	Definition/description
Residents	For one specific neighbourhood (LSOA), a resident is characterised as a user who has a home location within this LSOA in one day’s observation
Non-residents	For one specific neighbourhood (LSOA), a non-resident is characterised as a user without a home location within this neighbourhood in one day’s observation, or without home location detected
Residents’ movement behaviour variables*	Resident’s movement behaviour variables are a set of five categories to characterise the distance, entropy, and duration of this resident’s movement, including residents’ maximum distance from home (RMDH), residents’ radius of gyration (RRG), residents’ travelled distance (RTD), residents’ mobility entropy (RME), residents’ stay-at-home duration time (RSHDT)
Residents’ visiting behaviour variable**	For one LSOA, residents’ visiting behaviour variable is represented by residents’ footfalls (RF), counts of stays within this LSOA where the home location is situated
Non-residents’ visiting behaviour variable**	For one LSOA, non-resident’s visiting behaviour variable is represented by non-residents’ footfalls (NRF)

*LSOA- and month-level measurement can be found in Sect. 2.2.2

**LSOA- and month-level measurement can be found in Sect. 2.2.3

#### Resident and non-resident discrimination based on stay and home location detection

**Fig. 2:**
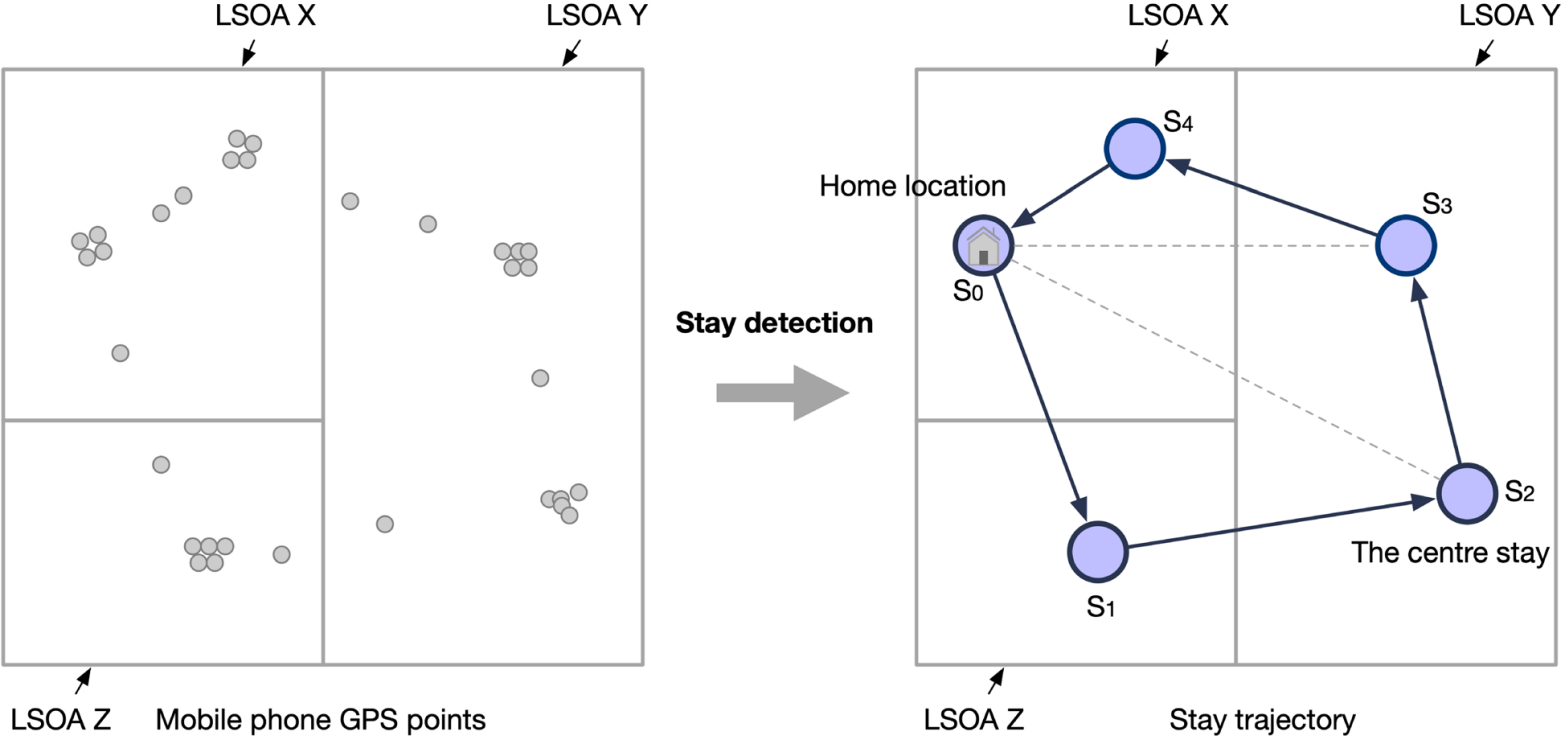
A user’s stay trajectory (across three LSOAs) generated from raw GPS points through stay detection. The grey boundaries are denoted as LSOA X, LSOA Y, and LSOA Z

*Stay definition*. A *stay* refers to a single user *u* spending some time at one location, where the user’s recorded GPS points are concentrated at or around the same location during the observed duration (Hariharan and Toyama 2004; Zheng 2015; Zhao et al. 2016). Figure 2 illustrates the process of detecting a user’s stay trajectory using several stay points from raw GPS data. Formally, a user’s raw GPS trajectory ${\textbf{P}}$ can be represented as a set of locations ${\textbf{l}}$ with temporal information, so each GPS point can be denoted as ${\textbf{P}_{i}} = (\textbf{l}_{i}, t_i)$. Given that a stay trajectory $\textbf{S}$ can be extracted from ${\textbf{P}_{i}}$, each stay can be represented as $\textbf{S}_{i}=\left( \textbf{l}_{i}, t_{i}^{\text{ start } }, t_{i}^{\text{ end } }\right)$.

In this analysis, the stay detection algorithm proposed by Hariharan and Toyama (2004) was implemented. This algorithm relies on two pre-defined parameters: $\Delta d$ – the maximum Euclidean distance that the recorded points of a user’s movement around a point/location to count as a stay, and $\Delta t$ – the minimum duration that the GPS records stay within time distance to qualify as a stay at that location. For this study, $\Delta d$ and $\Delta t$ were set to 50 meters and 5 minutes to delineate stays from the raw GPS trajectory data. The parameters are based on the assumption that stays identified from GPS points using these thresholds represent the typical range of an individual’s visits to a location, and have been commonly used in urban analytics (Zhao et al. 2015; Chen et al. 2023, 2025). For example, using a threshold of 5 minutes and 50 meters to define stays, Kang et al. (2005) demonstrated that these parameter settings effectively identify significant places from GPS location points. It is acknowledged, however, that this tests a single stay threshold and further research might explore the implications of this choice further.

*Home location delineation*. Using the semantic information in relation to human residence behaviour, we can infer the user’s home location based on the movement and stay pattern from their detected stay trajectory (Csáji et al. 2013; Phithakkitnukoon et al. 2012). In this analysis, a user’s (*u*) home location was defined as the detected stay location that the user visits the most frequently during the nighttime period of a day. Home location detection *h* can be described as:

1 $$h({\boldsymbol {S}};u)=arg\,\underset i{max}\left|\left\{{\boldsymbol S}_i\mid t_i^\mathrm{start},t_i^\mathrm{end}\in\left[t_{night\_begin},t_{night\_end}\right]\right\}\right|$$

For the purpose of this analysis, the night-time period is set to be from 11 PM to 6 AM for implementing the home location detection, i.e., one user’s home location is where a stay occurs most frequently from 11 PM to 6 AM during the stay trajectories in one day. By implementing stay detection and delineating home locations, it is possible to link each user’s home location to a neighbourhood area (represented by LSOAs in this study). Within the context of LSOAs, stays can be categorised either as those shared by residents or by non-residents within a given day. For example, the stays are labelled as a resident of LSOA X (see Fig. 2) because the home location ($S_0$) is identified in LSOA X though other stays of LSOA X although this resident does undertake stays in other LSOAs (here LSOA Y and Z) in a day. Thus, for LSOA X, stays made by residents (individuals whose home locations are in LSOA X) within LSOA X are labelled as ‘resident stays’ ($S_0$ and $S_4$ are the resident stays for LSOA X) while stays they make to other LSOAs are labelled as ‘non-resident stays’ ($S_2$ and $S_3$ are the non-resident stays for LSOA Y). In home location delineation, the mobile phone data was securely processed for safe storage and the home location information was eventually disposed after the completion of this work.

#### Measuring movement variables for residents

In terms of resident movement behaviours, longer routine trips may reflect reduced availability of capable guardianship at home (Felson et al. 2020; Tseloni et al. 2004). For example, when individuals travel further from their residence, they are absent for a longer duration, which weakens the routine guardianship and increases opportunities for residential burglary. In this regard, we employ several distance-based variables, such as maximum distance from home and total travelled distance to capture resident movements. Conversely, we also calculate the duration of time spent at home to reflect the resident’s presence at home. Following the movement distance measures that reflect guardianship in neighbourhoods, we also include the radius of gyration, which captures the extent of an individual’s habitual activity space. A larger radius indicates greater spatial dispersion of daily routines, which may weaken local guardianship by reducing time spent within the immediate neighbourhood. Similarly, we incorporate mobility entropy, which reflects the diversity and unpredictability of routine movements. High entropy indicates irregular schedules and spatial patterns with less predictable routines. Such irregularity may increase burglary risk by reducing the consistency of natural guardianship at home and it also makes guardianship level less predictable to offenders who might perceive this as an increased risk of discovery. Another implication is that these mobility behaviours are closely linked to the socioeconomic status of residents and may indirectly influence neighbourhood social control according to social disorganisation theory (Browning et al. 2020). Therefore, including these variables in our analysis allows us to examine the broader connections between different aspects of mobility behaviours and burglary levels.

In this analysis, the daily movement patterns of individual residents are measured based on their daily stay trajectories. Subsequently, the measurements for all residents across all LSOAs are aggregated at the LSOA- and month-level as collective mobility variables. There are some special variables that can be measured for resident movement, including stay-at-home duration time, the maximum distance from home, the travelled distance, mobility entropy and radius of gyration. Whilst measured from the home location ($S_0$), these movement variables reflect the resident’s whole daily mobility trajectory across the urban areas (i.e. they are not restricted to movement within the home LSOA). These are now defined in turn.

*Stay-at-home duration time*. A resident’s stay-at-home duration time (*hours*) is the total stay duration time length at the home location. This duration time (including the night-time period) can be measured from the detected home location in Sect. 2.2.1. In detail, based on an individual (*u*) home location’s (*h*) stay ($\textbf{S}_{h}=\left( \textbf{l}_{h}, t_{h}^{\text{ start } }, t_{h}^{\text{ end }}\right)$), one user’s (*u*) stay-at-home duration time ($\Delta _{t, h}$) can be calculated by $\Delta _{t, h} = t_{h}^{\text{ start }} - t_{h}^{\text{ end }}$.

*The maximum distance from home*. The maximum distance from home is the maximum value of the Euclidean distance between stays at the home location (Canzian and Musolesi 2015). For a resident’s stay trajectory $\textbf{S}$, the maximum distance from home $dh_{max}(\textbf{S}; u)$ is calculated as:

2 $$\begin{aligned} dh_{max}(\textbf{S}; u) = \max _{1\le i<n}\left| \textbf{S}_{i}, h(\textbf{S};u)\right| , \end{aligned}$$

where $\left| \textbf{S}_{i}, h(u)\right|$ is the Euclidean distance (*km*) between a stay $\textbf{S}_{i}$ and the home location $h(\textbf{S}; u)$ (see Eq. 1), considering *n* stays. For example, the maximum distance from home in Fig. 2 is between the home location $S_0$ and $S_2$ (i.e., $\left| S_0, S_2\right|$).

*Travelled distance*. This is the sum of the Euclidean distance between two consecutive (time-ordered) stays (Williams et al. 2015; Lu et al. 2012). For a user’s stay trajectory $\textbf{S}$, the travelled distance can be denoted as:

3 $$\begin{aligned} td(\textbf{S}; u) = \sum \limits _{i=1}^{n}\left| \textbf{S}_{i-1}, \textbf{S}_{i}\right| , \end{aligned}$$

where $\textbf{S}_{i-1}$ and $\textbf{S}_{i}$ are two successive stays in the *n* stays. In Fig. 2, the travelled distance can be calculated as $td = \left| S_0, S_1\right|$ + $\left| S_1, S_2\right| + \left| S_2, S_3\right| + \left| S_3, S_4\right| +\left| S_4, S_0\right|$.

*Radius of gyration*. The radius of gyration, as a radial distance to a point, is used to characterise the typical distance travelled by a centre stay (time-ordered) in the mobility trajectory (Gonzalez et al. 2008). Specifically, the radius of gyration is the root-mean-square distance of the object’s parts from the axis of rotation. For a user’s stay trajectory $\textbf{S}$, the radius of gyration is defined as:

4 $$\begin{aligned} rg(\textbf{S}; u) = \sqrt{\frac{1}{n} \sum \limits _{i=1}^{n} (\left| \textbf{S}_{i}, \textbf{S}_{m}\right| )^2 }. \end{aligned}$$

For example, the radius of gyration in the trajectory in Fig. 2 (the centre stay is $S_2$) can be calculated as $rg = \sqrt{\frac{1}{5} (\left| \textbf{S}_{2}, \textbf{S}_{0}\right| )^2 + (\left| \textbf{S}_{2}, \textbf{S}_{1}\right| )^2 + (\left| \textbf{S}_{2}, \textbf{S}_{3}\right| )^2+(\left| \textbf{S}_{2}, \textbf{S}_{4}\right| )^2}$.

*Mobility entropy*. The mobility entropy captures the full spatio-temporal order in an individual’s (*u*) mobility patterns (stay trajectories), which depends not only on the frequency of stays but also on the order in which the location nodes were visited and the time spent at each location (Song et al. 2010). The mobility entropy of an individual is defined as

5 $$\begin{aligned} E(u)=-\sum \limits _{T_u^{\prime }} P\left( T_u^{\prime }\right) \log _2\left[ P\left( T_u^i\right) \right] \end{aligned}$$

where $P\left( T_u^{\prime }\right)$ is the probability of finding a particular time-ordered sequence $T_u^{\prime }$ in the trajectory $T_u$. For the stay at a distinct location at *T*, the probability is determined by the fraction of the duration time an individual spent in the location divided by the total number of observations (i.e., 24 hours). Notably, mobility entropy measures the diversity of individual trajectories and higher entropy implies higher diversity with less predictability.

*Monthly daily average measurement of residents’ collective movement for LSOAs*. The prior measurements focused on an individual resident’s daily movement patterns. These can be aggregated to capture the collective movement behaviours (i.e., the five types of movement variables) of residents at the month level for each LSOA. The monthly daily average movement variable ($\bar{M}_{\text {monthly}}$) is defined as

6 $$\begin{aligned} \bar{M}_{\text {monthly}} = \frac{1}{D} \sum \limits _{d=1}^D \left( \frac{\sum _{i=1}^{N_d} M_{i,d}}{N_{d}} \right) \end{aligned}$$

where $\frac{\sum _{i=1}^{N_d} M_{i,d}}{N_d}$ is the calculation of daily mean movement variable per resident for one day observation (*d*) in a LSOA. Specifically, $M_{i,d}$ is the movement variable value for resident *i* on day *d* and $N_d$ is the total number of residents observed in the LSOA on day *d*. Then, $\bar{M}_{\text {monthly}}$ is the mean value of the movement variables over all days of the month ($\frac{1}{D} \sum _{d=1}^D$) in one LSOA. *D* is the total number of days of a month.

As a point of clarification, for each LSOA, this study does not include calculations related to the movement patterns of non-residents, primarily due to the complexities introduced by the dynamic shifts in population, e.g., a neighbourhood area (LSOA) can experience very large amounts of visiting from non-residents. Instead, this focus is solely on understanding how the range of movement behaviours of residents influences the neighbourhood’s effect on crime levels.

#### Measuring visiting variables for residents and non-residents

To measure the variables associated with visits in LSOAs, each day’s stays within the LSOAs are categorised into two types: stays by residents and stays by non-residents. The residents’ footfalls, as measured by a monthly daily average value, in a specific LSOA refer to the number of visits made by local residents whose primary residence is within that LSOA. In contrast, the non-resident footfall measured for an LSOA encompasses the visits made by individuals who are not local to the area. This non-resident population group includes residents from other LSOAs as well as visitors from beyond this study area (London) who do not have detected home locations within London in this analysis.

It is important to highlight that aggregated stays primarily focus on specific visitation or social activities at place services and venues. Consequently, certain types of stays unrelated to visitation patterns were intentionally excluded: (1) Stays occurring during the early morning hours (0 AM to 6 AM) are typically excluded, as these hours are characterised by minimal social activity and widespread business closures. Consequently, such stays are not considered representative of typical human mobility patterns (Traunmueller et al. 2018). (2) The user’s home location is defined as the place most frequently visited during nighttime hours (11 PM to 6 AM), reflecting habitual residential presence, so these stays are excluded (Pappalardo et al. 2016; Verma et al. 2024). (3) A user’s workplace is defined as any location where they remain for more than six consecutive hours between 7 AM and midnight. Work-related stays are distinguished from general visits and excluded, as they represent unique behavioural patterns that could otherwise bias the analysis. This method aligns with prior studies that have inferred workplace locations using mobile phone data (Yan et al. 2019).

Following this categorisation, for one LSOA, the accumulated stays of both residents (whose home locations are within this LSOA) and non-residents (whose home locations are not within this LSOA, but within other LSOAs, or without a home location detected) were first compiled to determine footfalls (or the counts of stays) at the LSOA level and daily level. Then, the monthly daily average footfall is introduced as a metric representing visitation variables.

In line with the approach used to measure movement variables for residents, we aggregated visiting variables for both residents and non-residents at the monthly level. This aggregation ensures consistency with the temporal resolution of the crime data employed in this analysis, which is available only at the monthly temporal scale. To clarify, calculating the monthly daily average footfall for a single LSOA involves firstly summing up the footfalls within the LSOA over a month. Subsequently, this sum is divided by the total number of days in the month to determine the monthly daily average value.

### Explainable machine learning models

#### XGBoost and SHAP

Explainable machine learning (ML) refers to methods and techniques in the field of artificial intelligence (AI) that offer insights into the impact of input predictors on outcomes of machine learning models (Molnar 2020). A machine learning model named XGBoost (short for ‘Extreme Gradient Boosting’) with an explainability technique known as ‘SHapley Additive exPlanations’ (SHAP) was selected for interpreting the impact of collective mobility variables on burglary incident levels in this study. XGBoost is a widely used machine learning algorithm valued for its efficiency and accuracy across diverse data types. It can manage multicollinearity effectively and is well-suited to capturing non-linear relationships within data as it employs an ensemble of tree-based models as base learners. It utilises gradient boosting machines (GBMs) to iteratively refine the predictions of multiple weak learners (decision trees) to enhance both accuracy and generalisation (Freund et al. 1999; Chen and Guestrin 2016). Furthermore, existing work demonstrates that XGBoost together with SHAP can also detect spatial effects in the data compared to traditional geostatistical models applied in urban analytics (Li 2022).

While traditional feature importance indices in tree-based models provide valuable insights, there are significant limitations in achieving full interpretability of the trained model. These limitations mainly arise from feature importance calculations that depend on heuristic methods, such as Gini importance or mean decrease impurity, which often inadequately reflect the complex interactions between input features. These feature importance methods often exhibit bias, particularly in their treatment of features with a higher number of categories. Furthermore, they fail to indicate the direction of a feature’s influence, leaving it unclear whether an increase in a feature’s value will positively or negatively affect the predicted outcome.

SHAP (Shapley Additive Explanations) proposed by Lundberg and Lee (2017) is a powerful tool for interpreting model outputs. By integrating the concept of game theory and local explanations (Štrumbelj and Kononenko 2014; Ribeiro et al. 2016; Shapley 1953), SHAP provides a systematic approach to quantify the contribution of each feature to the model’s predictions. The SHAP value for feature *i* represents the average contribution of feature *i* in the model’s prediction when it is added to different subsets of features, weighted by the probability of each subset forming before feature *i* is added. Thus, the SHAP value $\varnothing _i(v)$ for each feature *i* can be denoted as:

7 $$\begin{aligned} \varnothing _i(v)=\sum \limits _{S \subseteq N \backslash \{i\}} \frac{|S| !(n-|S|-1) !}{n !}(v(S \cup \{i\})-v(s)) \end{aligned}$$

Where *N* is the set of all features and *n* is the total number of features, *S* is a subset of features not including feature *i*, and *v* is the model function that gives the prediction for each subset of features. So, the $v(S \cup \{i\})-v(s)$ represents the prediction changes after we include the new feature *i* in the model and $\frac{|S| !(n-|S|-1) !}{n !}$ represents the associated weight (i.e., marginal contribution). Then, $\sum _{S \subseteq N \backslash \{i\}} \frac{|S| !(n-|S|-1) !}{n !}$ is the weight by summing up the weights from all possible subsets *S*.

Hence, an absolute SHAP value represents the magnitude or strength of the impact that a feature has on the model’s prediction compared to the baseline prediction. Specifically, a larger absolute SHAP value for a feature indicates its greater importance in influencing the model’s output compared to other input features. Positive SHAP values ($>0$) for a feature *i* suggest that higher values of this feature contribute to increasing the predicted dependent variable, indicating a positive impact on the model’s predictions. Conversely, negative SHAP values ($<0$) for a feature *i* imply that higher values of this feature contribute to decreasing the predicted dependent variable, signifying a negative impact on the model’s predictions.

#### Modelling procedures

The modelling process includes training and testing an XGBoost regression model using seven collective mobility variables (summarised in the first column of Table 2), a neighbourhood deprivation index, and burglary incident numbers. Subsequently, the SHAP approach is applied to analyse and interpret the impact of mobility variables on burglary levels across spatio-temporal units.

In the data preparation phase, *z*-score standardisation was applied to both the explanatory variable matrix *X* with a dimension of 4,835 LSOAs $\times$ 24 months $\times$ 8 explanatory variables and the response variable *y* (burglary incident counts) for both the training and testing sets. In the training and testing process, the training set covers the 19-month data from January 2020 to July 2021 (about 80% of the total dataset) and the testing set covers a period of five months from August 2021 to December 2021 (about 20% of the total dataset). This follows the ‘80/20’ rule commonly used in a standard machine learning training setup (Hastie et al. 2009). Then, performance metrics such as Root Mean Square Error (RMSE) and the coefficient of determination ($R^2$) were used for the trained XGBoost regressor. Specifically, $R^2$ illustrates the percentage of the variance in the target variable that the model accounts for, whereas RMSE measures the discrepancy between the model’s predictions and the actual values. A higher $R^2$ coupled with a lower RMSE signifies superior model performance.

During the training phase of the XGBoost regressor, gradient boosting iteratively builds a collection of decision trees by minimising the cost function at each step. For hyperparameter tuning of the XGBoost regressor, grid search and cross-validation were employed to optimise the parameter settings defining decision trees, then the assembled XGBoost regressor (with the maximum tree depth, a sub-sample ratio of columns when constructing each tree, and learning rate) was selected by using 10-fold cross-validation in GridSearchCV^5^.

## Results

By analysing mobile phone data from 1,979,081 users, 1,055,438 residents were identified (the user obtains a home location) in London from 2020 to 2021. The initial step (described in Sect. 2.2.1) identified the home locations for distinguishing residents (who have detected home locations in London LSOAs) and non-residents (who can be either residents from other LSOAs of London or visitors from outside areas of London who do not have a detected home location in London LSOAs) for each LSOA on a daily basis. Subsequently, collective movement variables (including RMDH, RRG, RTD, RME and RSHDT shown in Table 1) were measured from each resident’s stay trajectory and visiting variables were aggregated to footfalls shared by residents and non-residents in LSOAs. Next, monthly daily average measurements were computed for 4,835 LSOAs over 24 months to generate the collective mobility (movement and visiting) variables at the LSOA- and month-level (see Table 2).

Following this, the XGBoost regression model was trained (with a maximum tree depth of 17, a learning rate of 0.02, and a sub-sample ratio of columns when constructing each tree of 0.9). The best model performance metric was an RMSE of 0.79 and $R^2$ of 0.66 using the given 19-month training dataset. During the testing phase, model performance metrics (RMSE of 1.19 and $R^2$ of 0.42) were determined by comparing the predicted burglary levels from the trained XGBoost regressor to the actual burglary levels in the 5-month dataset. Lastly, the SHAP strategy was applied to explain the optimised XGBoost model for both the training and testing sets by measuring the impact of the collective mobility variable levels on burglary levels from global and local perspective.

In this section, Sect. 3.1 describes the variations in collective mobility (movement and visiting behaviours) of both residents and non-residents in London LSOAs over 24 months. Subsection 3.2 describes the global impact of the collective mobility on burglary levels in different neighbourhoods including an exploration of how this changed over the COVID pandemic period. Subsection 3.3 then outlines how the model interprets local impacts by examining how the collective mobility variables influence burglary levels in specific LSOAs and months.

### The shifting of collective mobility in London from 2020 to 2021

In analysing the explanatory variables summarised in Table 2 (such as movement and visiting behaviour and neighbourhood disadvantage variables) and the dependent variable (burglary incident levels), it is important to note that in some cases the minimum count of both resident and non-resident footfall traffic (i.e., RF and NRF) drops to zero. This indicates a lack of visiting activities within a particular spatial-temporal unit/grid (i.e., one LSOA in one month) of 4,835 LSOAs across the 24 months. Table 2 also indicates that burglary is a sparse variable with a mean value of less than 1 in each LSOA per month.

**Table 2:** The description of explanatory variables (i.e., movement and visiting and neighbourhood deprivation) and dependent variable (burglary numbers) of 4,835 LSOAs and 24 months. All variables are measured at the LSOA level

Variables	Mean	Std	Min	Max
Residents’ maximum distance from home (RMDH) (km)	4.18	2.38	0.36	192.77
Residents’ radius of gyration (RRG) (km)	1.74	0.94	0.15	46.72
Residents’ travelled distance (RTD) (km)	9.14	4.72	1.11	386.81
Residents’ mobility entropy (RME)	2.21	0.32	0.63	3.74
Residents’ stay-at-home duration time (RSHDT) (hour)	7.6	1.55	0.70	14.06
Residents’ footfalls (RF)	5.66	8.91	0.00	920.52
Non-residents’ footfalls (NRF)	58.12	142.91	0.00	17368.10
Index of multiple deprivation (IMD)	21.50	10.91	0.00	64.70
Burglary incident numbers	0.98	1.35	0.00	40.00

**Fig. 3:**
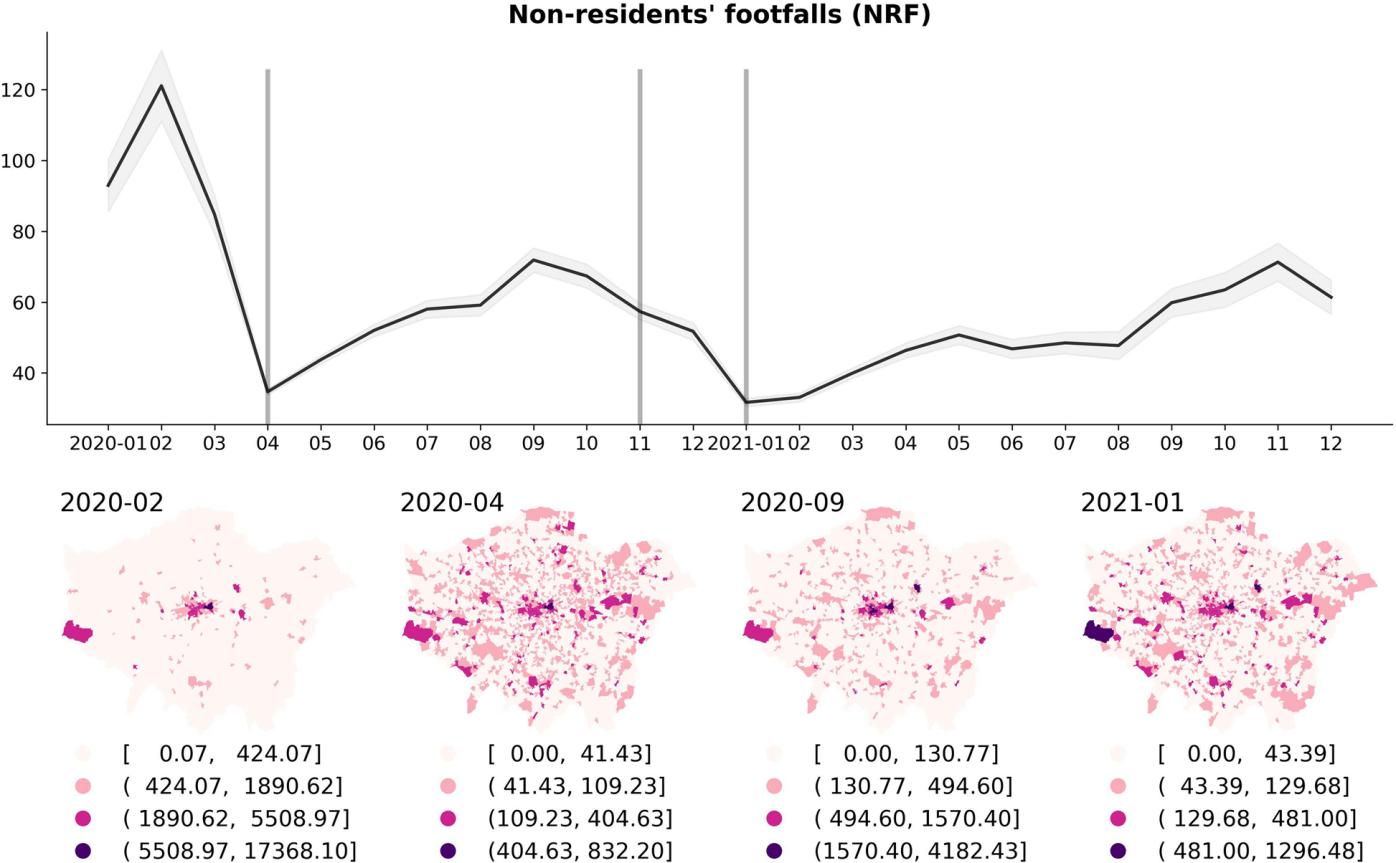
The temporal change (monthly) of ‘Non-residents’ footfalls’ (per LSOA) from 2020 to 2021, and the spatial distribution in selected four distinct restriction/relaxation months in London: February 2020 –‘Normal times/before lockdown’, April 2020 – ‘First national lockdown’, September 2020 – ‘First lockdown restrictions eased’, and January 2021 – ‘Third national lockdown’. Three vertical grey lines denote the specific national lockdown months in the UK, including the ‘First national lockdown’ (from March 23, 2020 to June 23, 2020), ‘Second national lockdown’ (from November 5, 2020 to December 2, 2020) and ‘Third national lockdown’ (started from January 6, 2021 to February 22, 2021)

Figure 3 shows the temporal change (monthly) of ‘Non-residents’ footfalls’ (per LSOA) from 2020 to 2021, and the spatial distribution in four distinct pandemic restriction/relaxation months during the periods of ‘Normal times/before lockdown’ (February 2020), ‘First national lockdown’ (April 2020), ‘First lockdown restrictions eased’ (September 2020), and ‘Third national lockdown’ (January 2021) in London. The average footfalls of non-residents (NRF) in London’s LSOAs experienced a decrease during restriction periods such as April 2020 and January 2021, while it increased during relaxation periods such as September 2020. Regarding the spatial dynamics of non-residential footfall, a discernible shift can be observed in the location of the areas with high-volume footfall across London LSOAs over the pandemic period. High volume footfall shifts from the city’s central regions to its peripheral urban areas from months under normal circumstances to the first national lockdown in the context of the overall decline in citizen activities (A similar pattern of local residents’ footfalls can also be found in Fig. 16 of Appendix A).

**Fig. 4:**
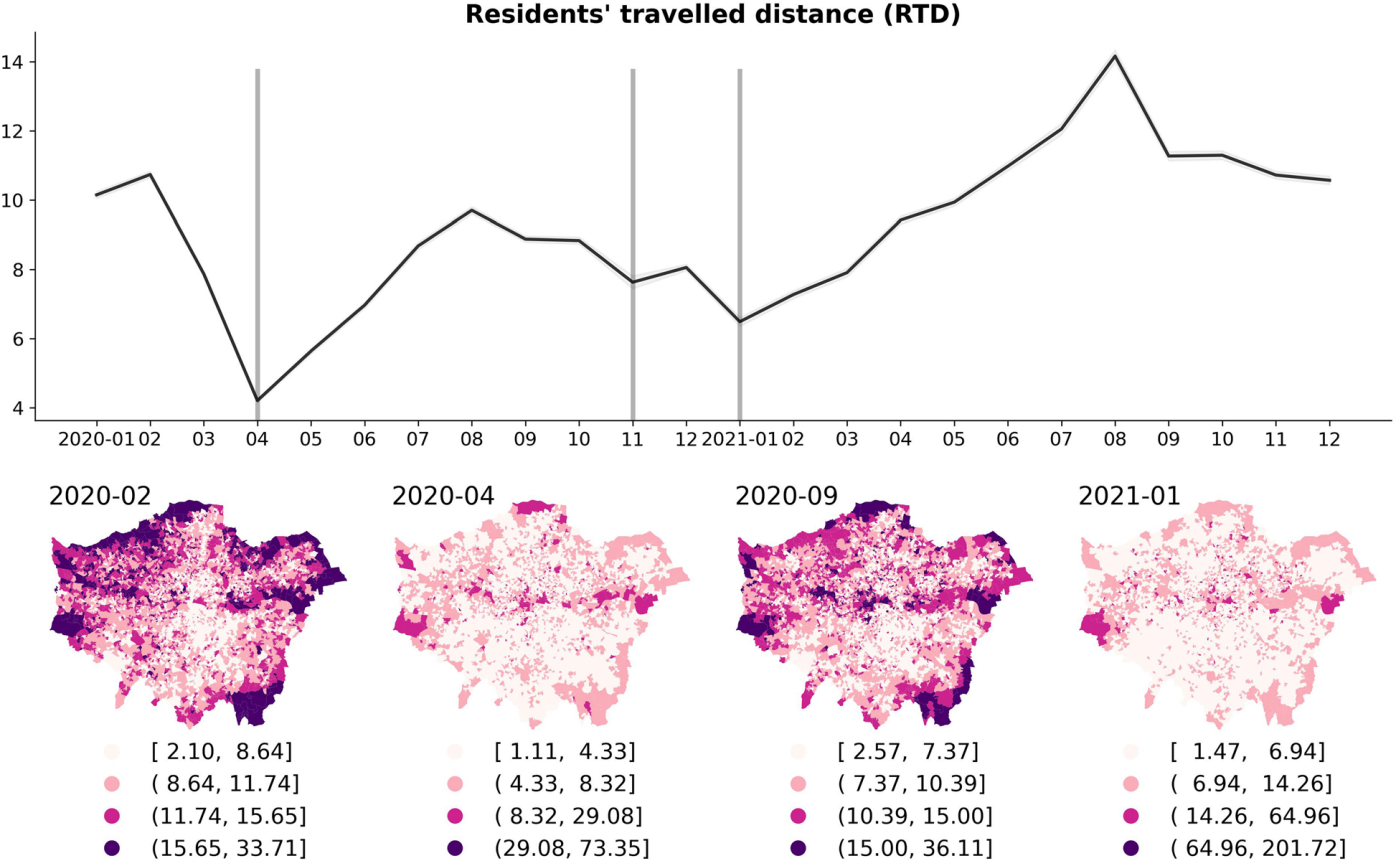
The temporal change (monthly) of ‘Residents’ travelled distance’ (per LSOA) from 2020 to 2021, and the spatial distribution in the selected four distinct restriction/relaxation months in London

The fluctuations in footfalls correlate with pandemic policy adjustments and there are similar temporal patterns observed in movement behaviours of local residents as indicated in Fig. 4. This demonstrates a change in the average travel distance of residents during distinct months coinciding with the lockdown policy. Furthermore, the maps in Fig. 4 show that residents living in the peripheries of London typically travel greater distances than those in central London during normal times (e.g., February 2020). However, the travelling distances of residents living in outer London decreased notably and some indicated shorter travelling distances than the residents in inner London during the lockdown month (e.g., April 2020).

**Fig. 5:**
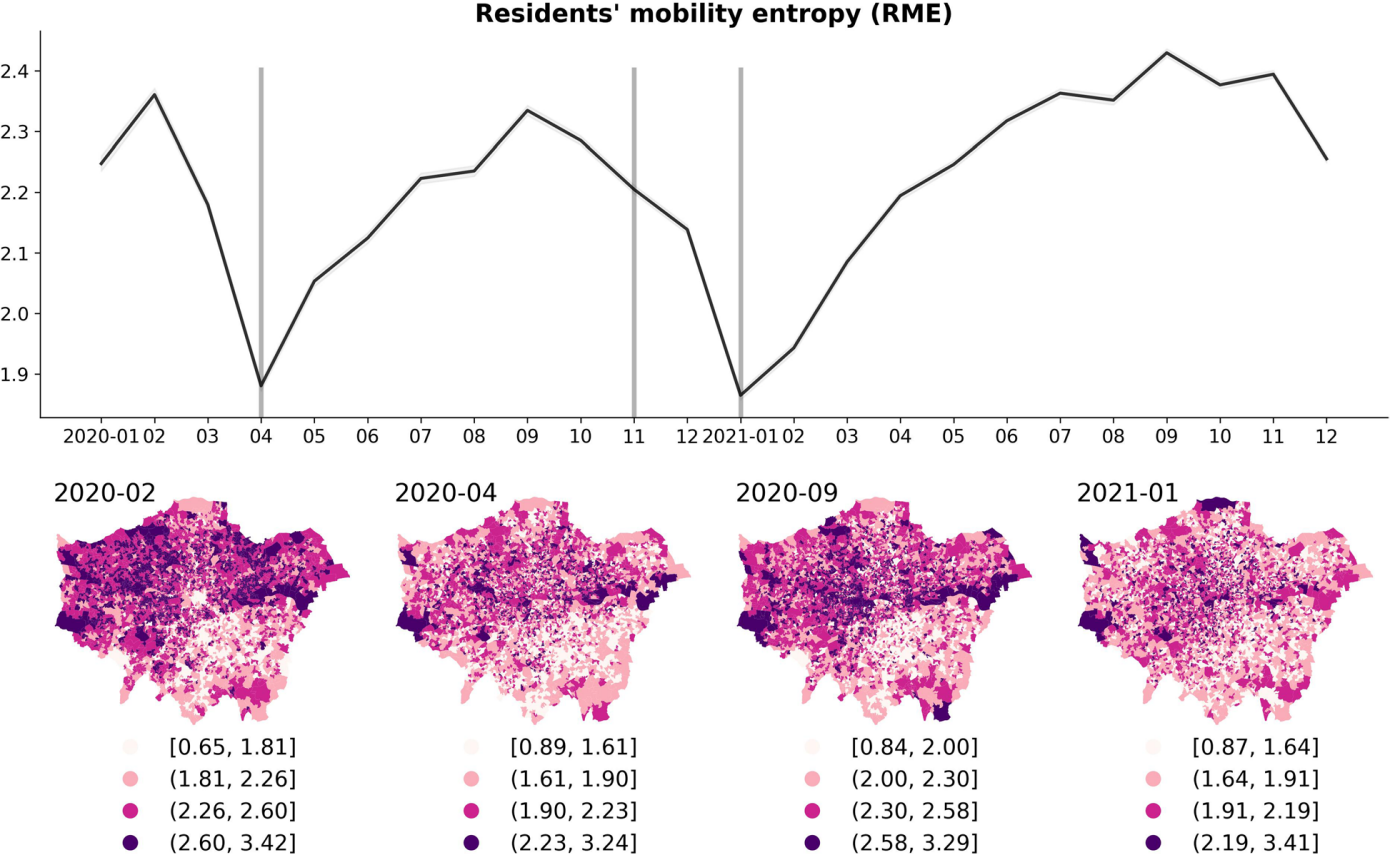
The temporal change (monthly) of ‘Residents’ mobility entropy’ (per LSOA) from 2020 to 2021, and the spatial distribution in the selected four distinct restriction/relaxation months in London

In line with this, the residents’ mobility entropy (representing movement diversity –see Fig. 5) also demonstrates a decrease during periods of restriction and an increase during relaxation times (other movement variables can be found in Appendix A: ‘Residents’ maximum distance from home’ shown in Fig. 17 in Appendix and ‘Residents’ radius of gyration’ shown in Fig. 18 in Appendix). Conversely, the residents’ stay-at-home duration time (shown in Fig. 19 in Appendix A) exhibited a marked spike during lockdown periods and reached its lowest level during relaxation phases, reflecting adjustments in working and social activities in response to policy changes.

### Global impacts of collective mobility on burglary

Utilising the SHAP strategy for interpreting the trained XGBoost model in conjunction with the explanatory variables, the generated SHAP values indicate the influence of collective mobility variables and neighbourhood disadvantage variable (IMD) on burglary levels across 4,835 $\times$ 24 spatio-temporal observational units/grids.

**Fig. 6:**
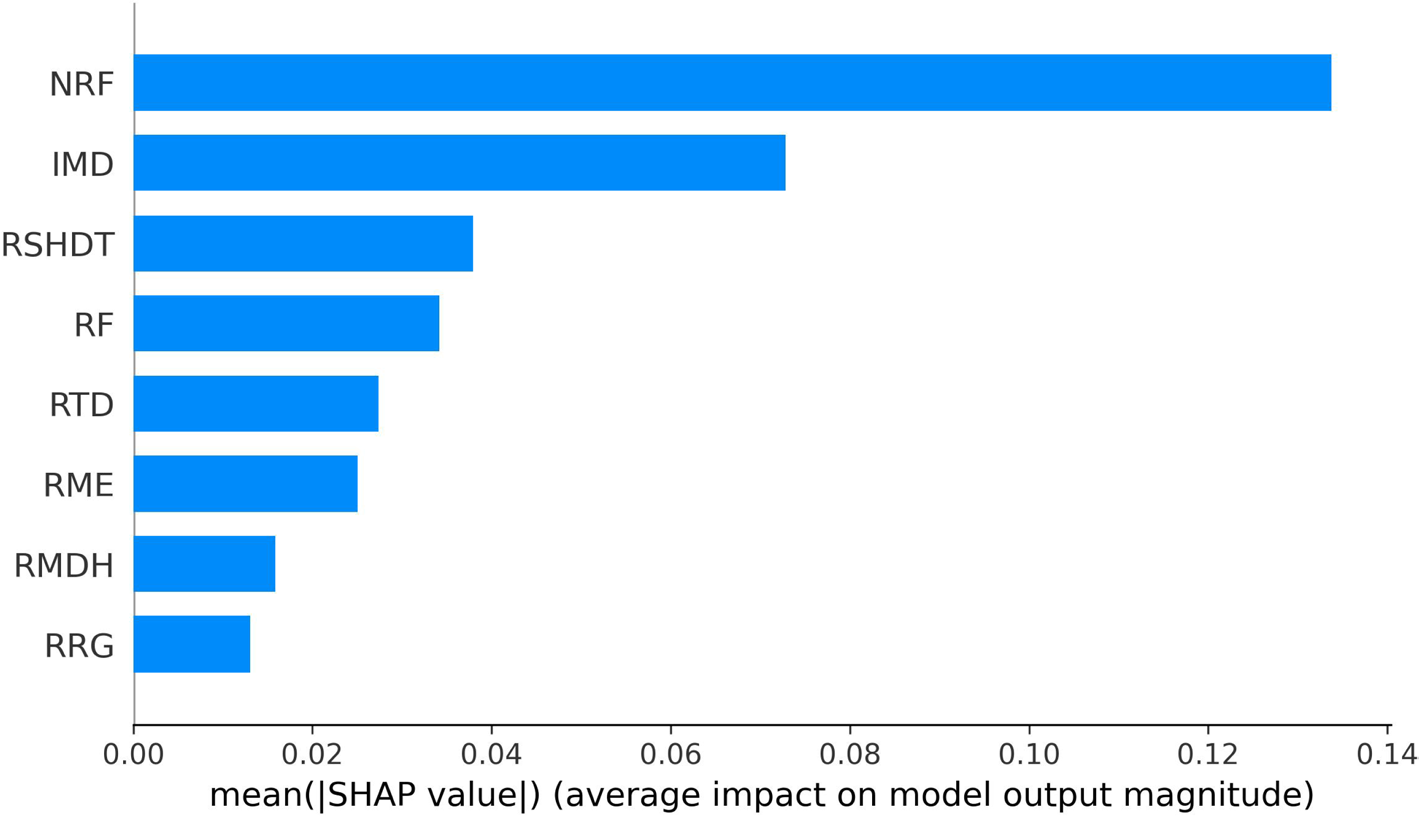
The impact of each explanatory variable (measured by the average absolute SHAP values of all months and LSOAs) on burglary levels in the trained XGBoost model

In Fig. 6, the global impacts quantifying the influence of each explanatory variable on the burglary level are measured by the average absolute SHAP values of the corresponding variable in all LSOAs and months from the optimised XGBoost regression model. It can be observed that the footfall traffic from non-residents (i.e., non-residents’ footfalls) obtained the highest value followed by the neighbourhood disadvantage variable (i.e., IMD), while the duration of residents’ time spent at home (RSHDT) ranked third in its influence on burglary levels in neighbourhoods. Additionally, residents’ movement variables (e.g., residents’ travelled distance (RTD), residents’ mobility entropy (RME), residents’ maximum distance from home (RMDH) and residents’ radius of gyration (RRG)) take limited influence on burglary levels.

To test for the potential impact of multicollinearity, we include the correlation matrix (see Fig. 14 in Appendix) and the variance inflation factors (VIF) (see Fig. 15 in Appendix) of the collective mobility variables in Appendix A. The results are mixed. The movement variables were found to be correlated based on Pearson correlation. IMD, however, is not strongly correlated with any of the variables across all samples. We also provide the VIF results, which indicate that RMDH and RRG exhibit high multicollinearity. However, in our XGBoost model, the SHAP values of these two variables show only minimal impact on the model output (see Fig. 6). This suggests that XGBoost is able to handle multicollinearity in the data while capturing the nonlinear relationships among variables. Although highly correlated variables can sometimes influence linear regression model outputs, in our case, their effect is limited in XGBoost.

**Fig. 7:**
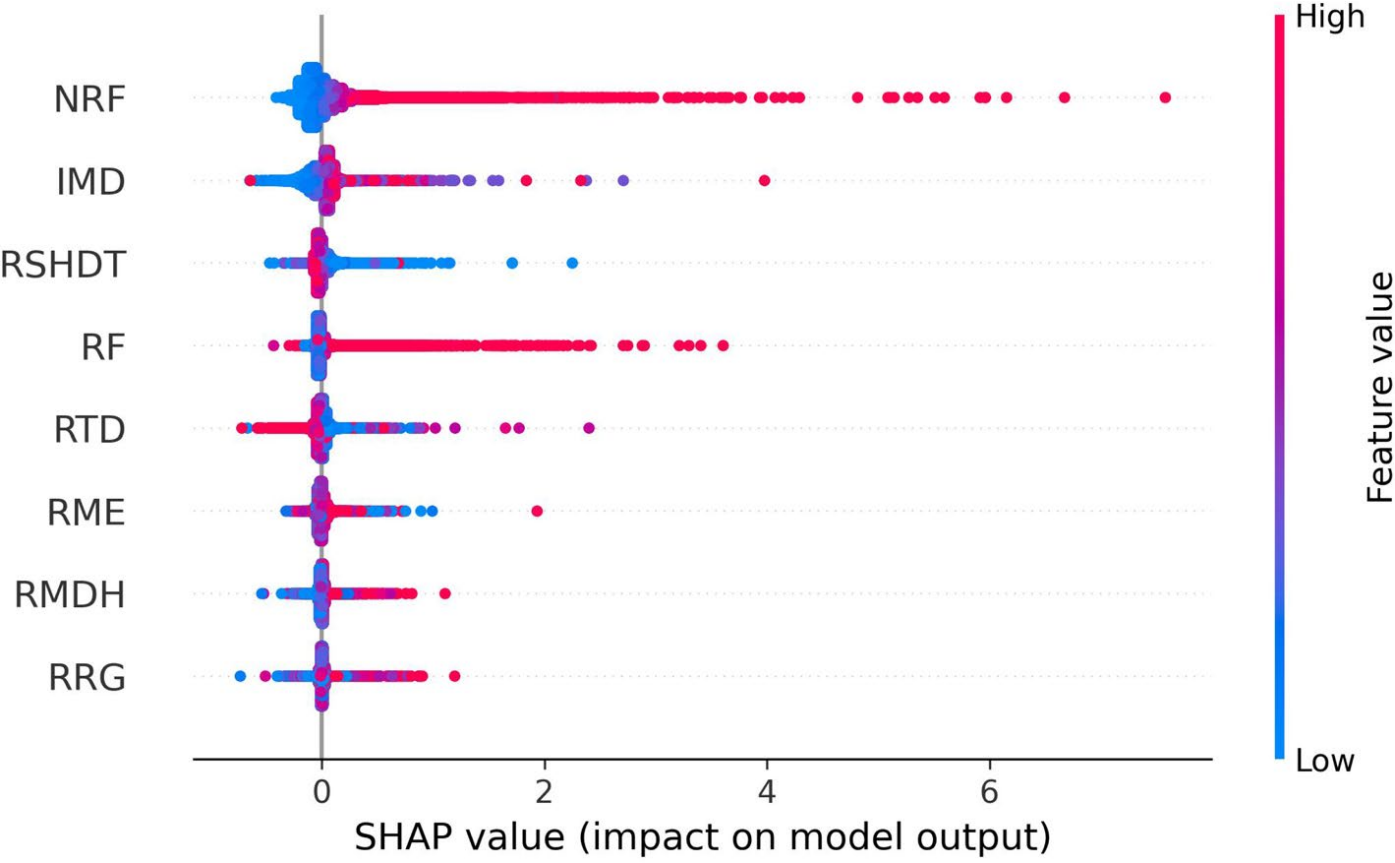
The distribution of SHAP values of each explanatory variable. The x-axis represents the levels of SHAP values. Values greater than 0 indicate a positive impact, while values less than 0 suggest a negative impact. In the legend, different levels of feature values, including mobility and neighbourhood disadvantage variables, are represented by colour variations: red signifies a higher level, while blue indicates a lower level

In Fig. 7, the distributions of SHAP values in all spatio-temporal observation units are systematically plotted. Recall that positive SHAP values show a positive impact on burglary (i.e., larger positive SHAP values mean increased levels of burglary). For each explanatory variable (feature), the red colour signifies higher values and the blue colour represents lower values of the mobility and disadvantage variables. The figure illustrates the global correlations between different levels of explanatory variables and their impacts (measured by SHAP values) on the burglary levels in all observation units/grids consisting of 4,835 LSOAs and 24 months. For example, higher values of NRF (non-residents’ footfalls) correlate with a pronounced increased in burglary levels, as demonstrated by the cluster of red dots to the right. A similar pattern is also observed with Index of Multiple Deprivation (IMD), where higher levels of IMD correspond to increased burglary levels. In contrast, RSHDT (residents’ stay-at-home duration time) imposes a negative impact on burglary levels, i.e., higher values of RSHDT are related to decreased crime levels (and vice versa, lower values of RSHDT are associated with an increase in crime levels).

**Fig. 8:**
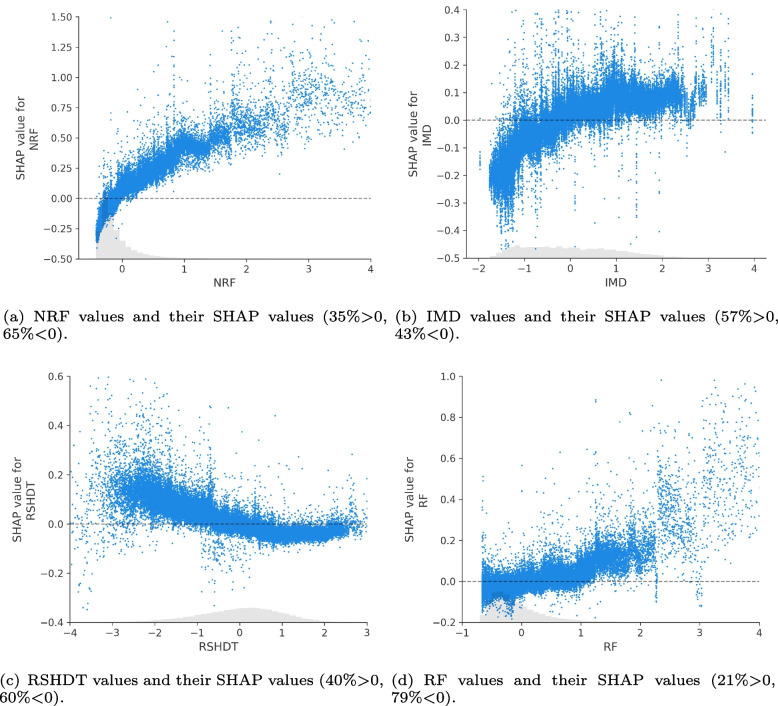
The dependency plot of the top four explanatory variable values (standardised) and corresponding SHAP values with proportions above and below zero

To disentangle how the explanatory variables contribute to the strength of impact on the burglary outcomes, Fig. 8 indicates the top four selected explanatory variable values (standardised values) and corresponding SHAP values in the majority of all samples (by excluding the outline sample points in the figure) from 4,835 LSOAs and 24 months (The figures of the SHAP values and features values for all samples can be found in Fig. 20 of Appendix A). It can be observed that the higher NRF values (as illustrated in sub-figure 8a) generally relate to a higher SHAP value. Further, negative SHAP values of NRF (i.e., below zero) are mainly found in low-level NRF values. Similarly, in sub-figure 8b, the higher SHAP values of IMD are generally correlated with higher IMD values.

In contrast, higher RSHDT (residents’ stay-at-home duration time) values tend to correlate with lower SHAP values shown in sub-figure 8c. In terms of the positive or negative influences of RSHDT on burglary levels in the observed samples, the inflexion point is observed approximately at 0 of the RSHDT standardised value (the real RSHDT value is about 3.8 hours). Further, the RF (residents’ footfalls) shown in sub-figure 8d did not demonstrate a significant correlation where higher RF values are associated with increased SHAP values, except when the RF value is above approximately 1 (true footfall value is 14.4), indicating that there might be a threshold at which resident footfall has an impact.

**Fig. 9:**
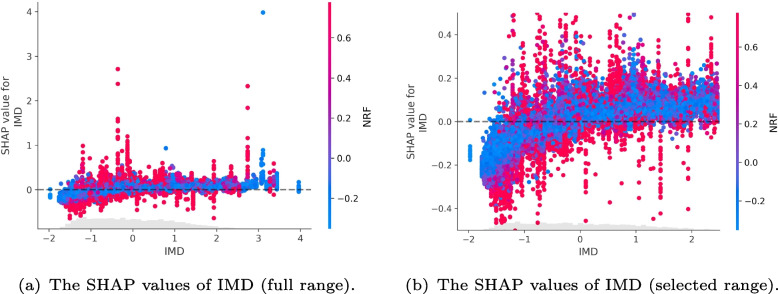
The SHAP values of IMD alongside IMD values with interactional NRF (non-residents’ footfalls) values. The red dots denote the high NRF values, while the blue dots denote the low NRF values

To demonstrate the interplay of different variables on burglary levels, Fig. 9 presents an example illustrating the impacts of the interaction between neighbourhood deprivation (IMD) and non-residents’ footfalls (NRF) on burglary levels. It shows the SHAP values for the IMD variable in different spatial-temporal units categorised by the corresponding level of the NRF values (higher levels of non-residents’ footfalls are coded as red while lower ones are blue). It is evident that the marginal blue dot in the top-right blue of sub-figure 9a indicates a high impact of IMD at elevated levels of deprivation in the context of a lower level of non-residents’ footfalls. Such interplay shows that the low-level non-resident visiting footfalls also can have a high and positive impact on burglary levels in a neighbourhood (LSOA) with high-level deprivation. Further, sub-figure 9b (selected range of IMDs) reveals a prevalence of red dots representing high-level NRF in less deprived neighbourhoods (i.e., the range below −1 on the x-axis of IMD values), that are exhibiting negative SHAP values (specifically, below zero). This means the high-level non-resident footfalls and less deprivation can contribute to an overall negative impact on burglary levels (i.e., decrease the burglary) in the neighbourhoods.

**Fig. 10:**
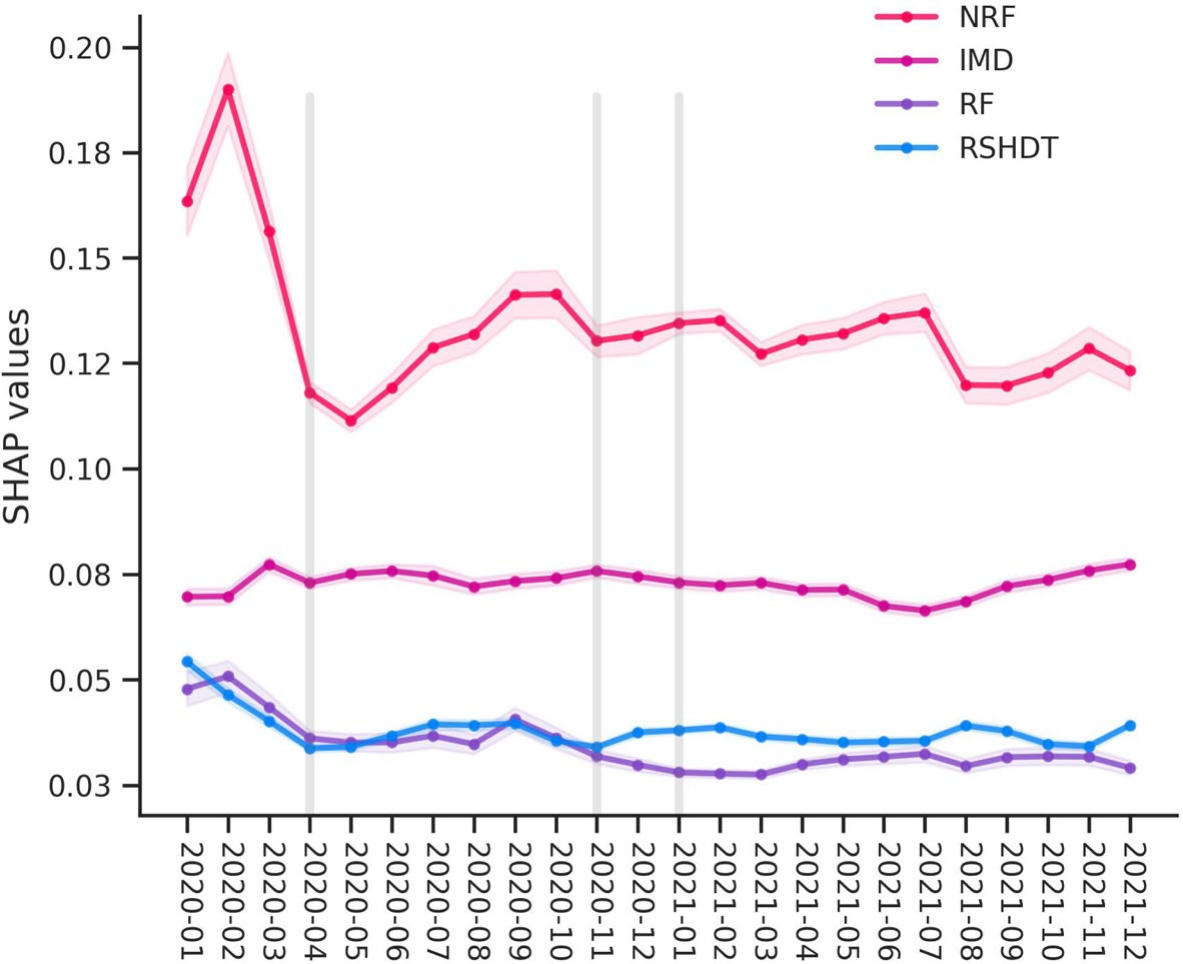
The monthly variation of average absolute SHAP values (per LSOA) of four selected variables (i.e., NRF, IMD, RSHDT and RF) during 24 months. The vertical lines denote the three distinct national lockdown periods

By measuring the average absolute SHAP values per LSOA, Fig. 10 shows that the global impacts of the top four variables on burglary levels are modulated by pandemic-related policies during the 24 months observation period in London LSOAs. For instance, the SHAP values of NRF show a lower relative impact of NRF on burglary during the first national lockdown period (e.g., April 2020 and May 2020) while it recovered to a higher impact level in the relaxation period (e.g., September 2020) followed by a second reduction during the second national lockdown (e.g., November 2020). Figure 10 also shows that the SHAP values of residents’ footfalls follow a similar pattern of fluctuation in impact, compared to no significant change in the impact of IMD on burglary during the 24 observed months. Examining the SHAP values for RSHDT, a difference can be observed in the SHAP values in response to restriction periods. Specifically, there was a decline in the SHAP values during the first national lockdown phase, followed by an increase subsequent to the second national lockdown period commencing in November 2020.

### Local impacts of collective mobility on burglary

**Fig. 11:**
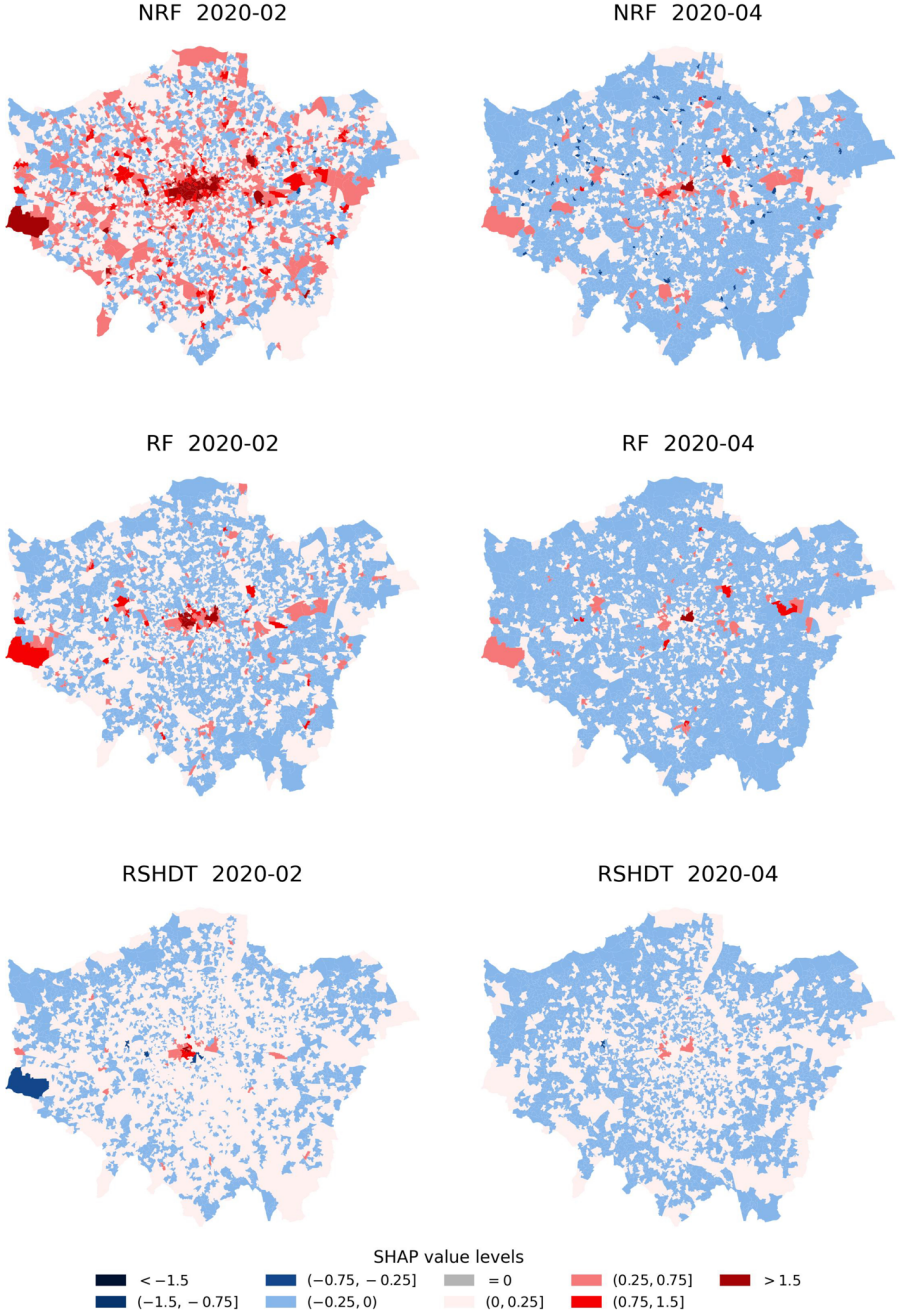
The distribution of the SHAP values of three selected variables (NRF, RSHDT and RF) during the ‘Before lockdown’ (represented by February 2020) and ‘First national lockdown’ (represented by April 2020) periods

The localised impacts of the population’s movement and mobility on burglary levels predominantly examine these influences at the neighbourhood area level (LSOA level) in London. By mapping the distribution of the SHAP values of three selected variables (NRF, RSHDT and RF) for the ‘Before lockdown’ (represented by February 2020) and ‘First national lockdown’ (represented by April 2020) periods, Fig. 11 denotes the difference across the selected variables in the different contexts of pandemic policy shifts. In an examination of the spatial distribution in London before the lockdown, a concentration of high and positive SHAP values (i.e., large positive impact on burglary) of NRF (plotted as red areas) was observed within the city centre surrounded by other dispersed high and positive value areas (see the map of ‘NRF 2020-02’). Conversely, during the first national lockdown (see the map of ‘NRF 2020-04’), the negative SHAP values (plotted as blue areas) dominated the majority of the urban areas, demonstrating a reversal in relationships (between NRF and burglary levels) excluding the city centre areas. Notably, several urban regions (e.g., western areas) were identified where high and positive SHAP values of NRF (high positive impact on burglary levels) persisted even during this period.

In the distribution of SHAP values of RF and RSHDT, the high and positive SHAP values (above 1.5) were discernibly concentrated within urban centres in normal times (see the map of ‘RF 2020-02’ and ‘RSHDT 2020-02’). During the first lockdown, though there was a noticeable reduction in SHAP values across the urban areas, the high and positive SHAP values of RF and RSHDT remained clustered in the same (inner city) areas. Distinctive variations in the distribution of SHAP values of explanatory variables are also evident in the three selected variables (NRF, RF and RSHDT) during the ‘Lockdown easing period’ (as exemplified by September 2020) and the ‘Second national lockdown’ (as exemplified by November 2020) periods (see Fig. 21 in Appendix A).

**Fig. 12:**
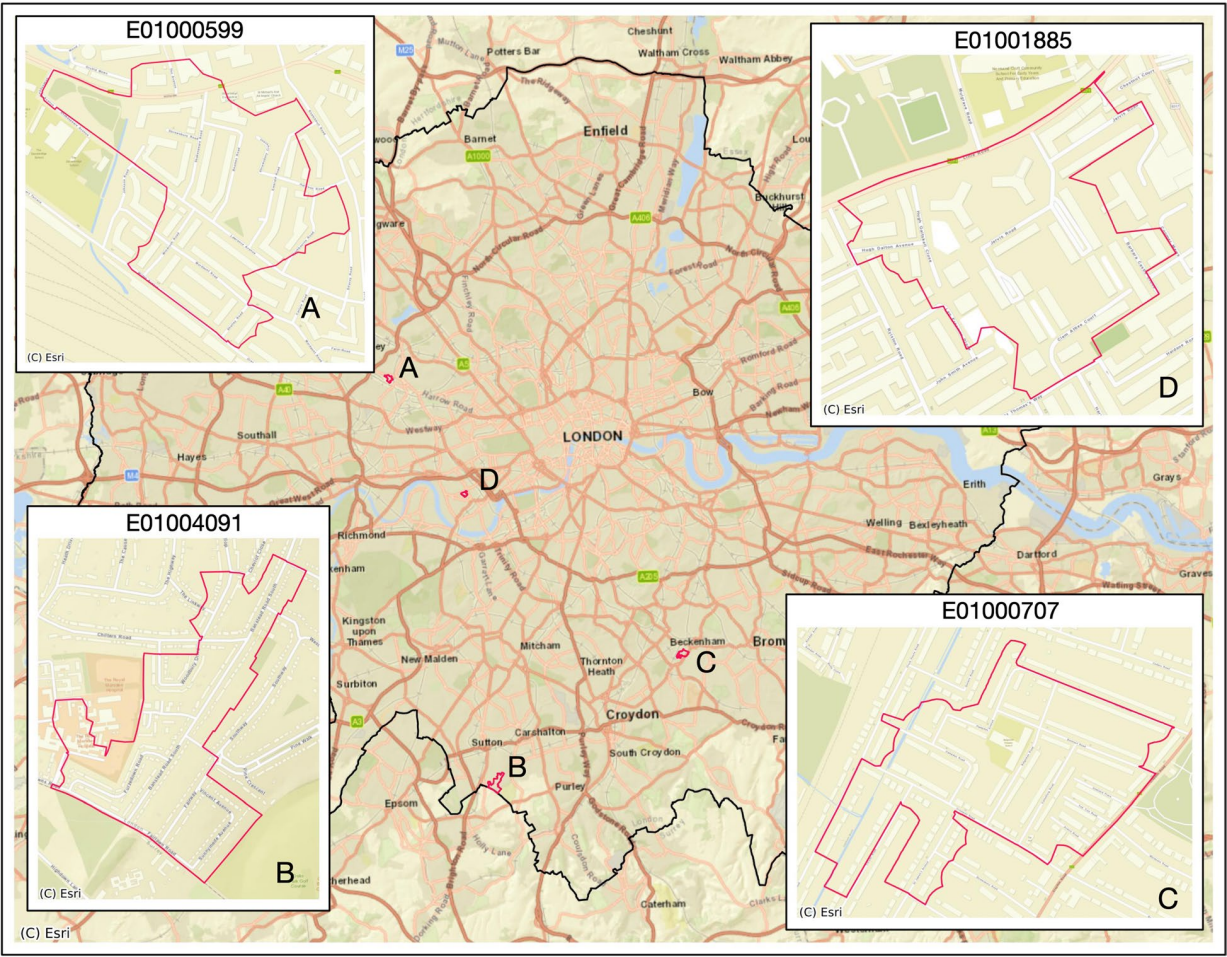
The map of four selected LSOAs in London

**Fig. 13:**
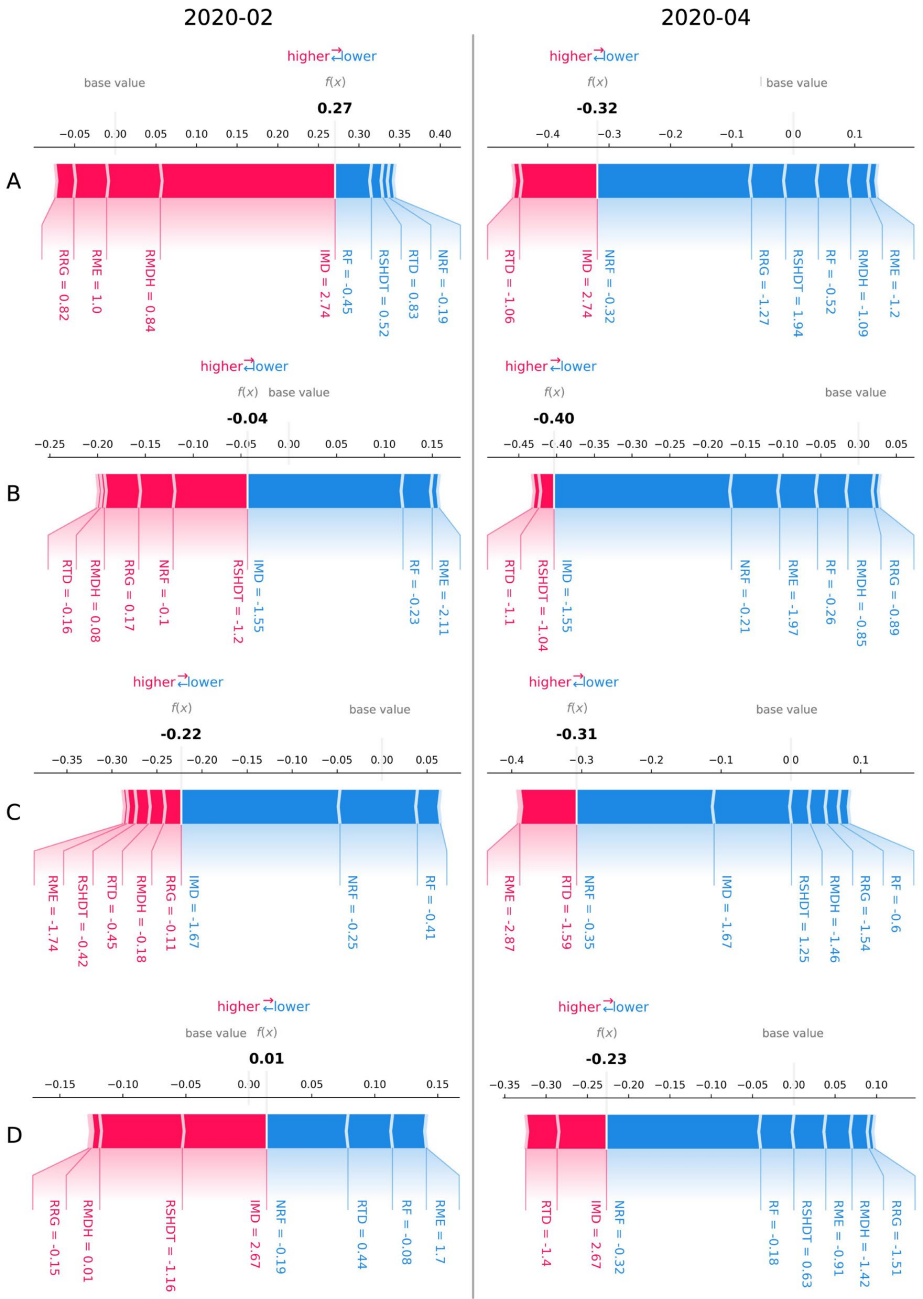
The SHAP values of contributed explanatory variables of four selected LSOAs in February 2020 (‘Before lockdown’) and April 2020 (‘First national lockdown’). The length of the vertical bar represents the magnitude (measured by the SHAP absolute value) of the contribution/impact of each variable on burglary-level prediction. A longer bar indicates a stronger impact and vice versa. The colour indicates the direction of the variable/feature’s impact on the prediction of burglary levels: red shows a positive (increased) impact while blue shows a negative (decreased) impact on the burglary level prediction. The standardised values of each variable are labelled under their respective vertical bars. The dark value under f(x) represents the burglary level predicted by the trained XGBoost model (note that this is different from the observed burglary levels). The base value refers to the mean of all predicted burglary levels across all samples, which in this study is 0.000038

**Fig. 14:**
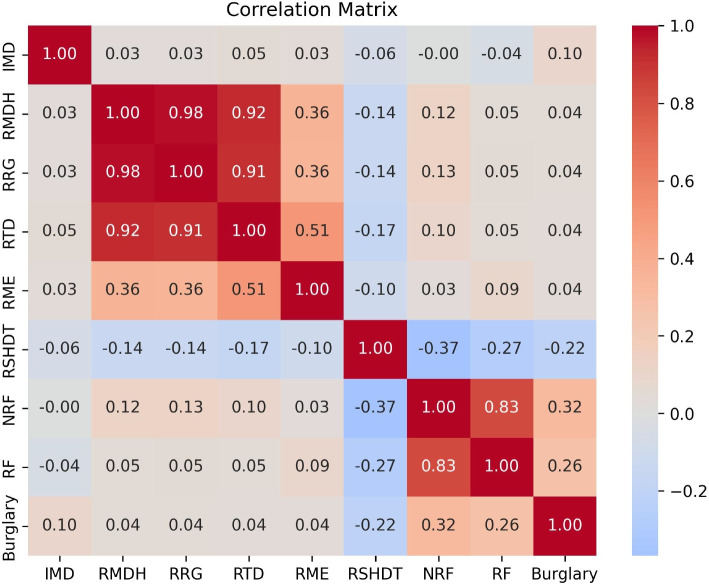
The correlation matrix of explanatory variables (collective mobility and IMD) and burglary variables

**Fig. 15:**
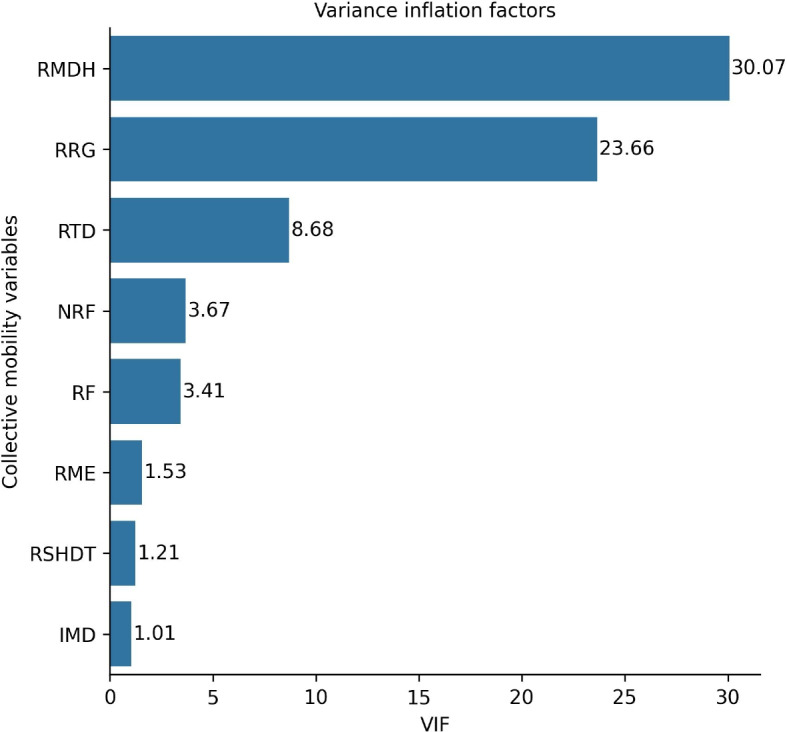
The variance inflation factors (VIF) of collective mobility variables and IMD

**Fig. 16:**
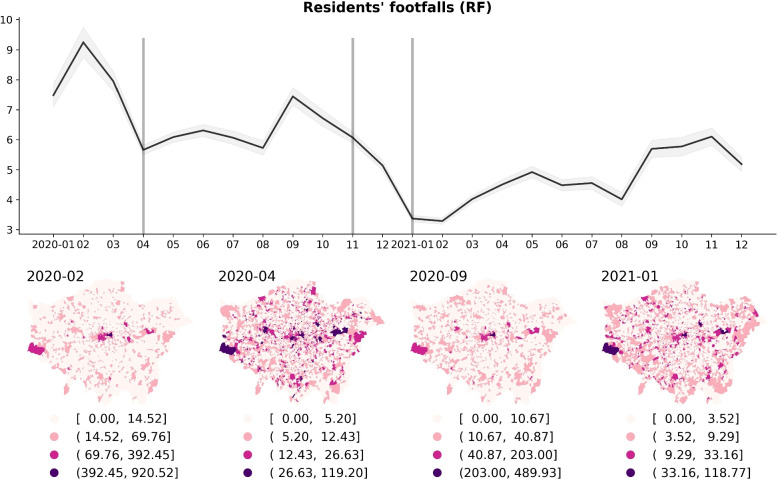
The temporal change (monthly) of local residents’ footfalls (per LSOA) from 2020 to 2021, and the spatial distribution in the selected four distinct restriction/relaxation months in London

**Fig. 17:**
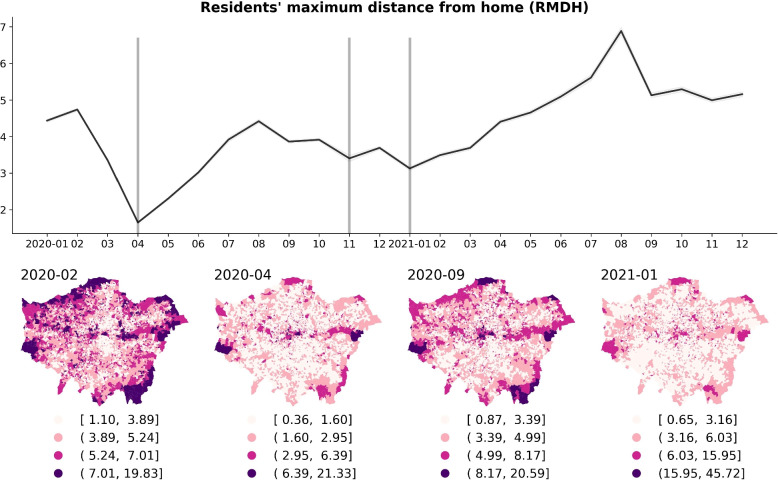
The temporal change (monthly) of residents’ maximum distance from home (per LSOA) from 2020 to 2021, and the spatial distribution in the selected four distinct restriction/relaxation months in London

**Fig. 18:**
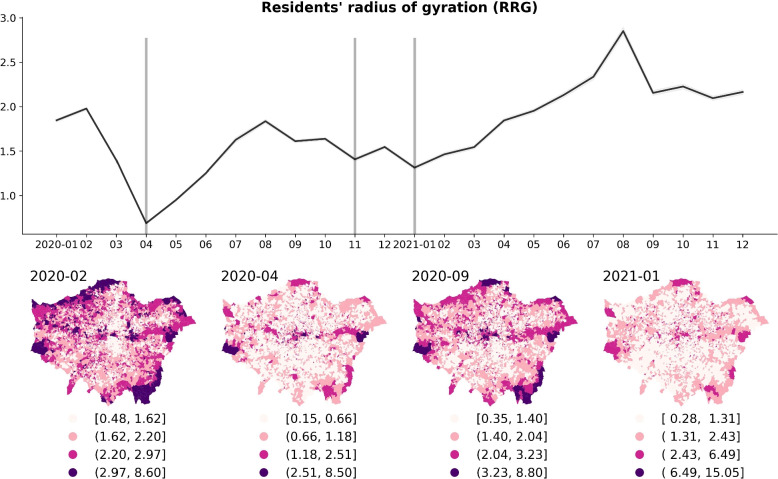
The temporal change (monthly) of ‘Residents’ radius of gyration’ (per LSOA) from 2020 to 2021, and the spatial distribution in the selected four distinct restriction/relaxation months in London

**Fig. 19:**
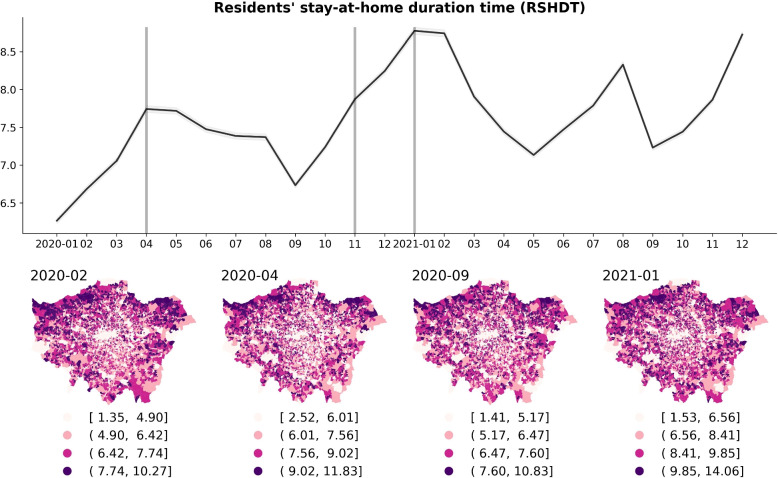
The temporal change (monthly) of ‘Residents’ stay-at-home duration’ (per LSOA) from 2020 to 2021, and the spatial distribution in the selected four distinct restriction/relaxation months in London

**Fig. 20:**
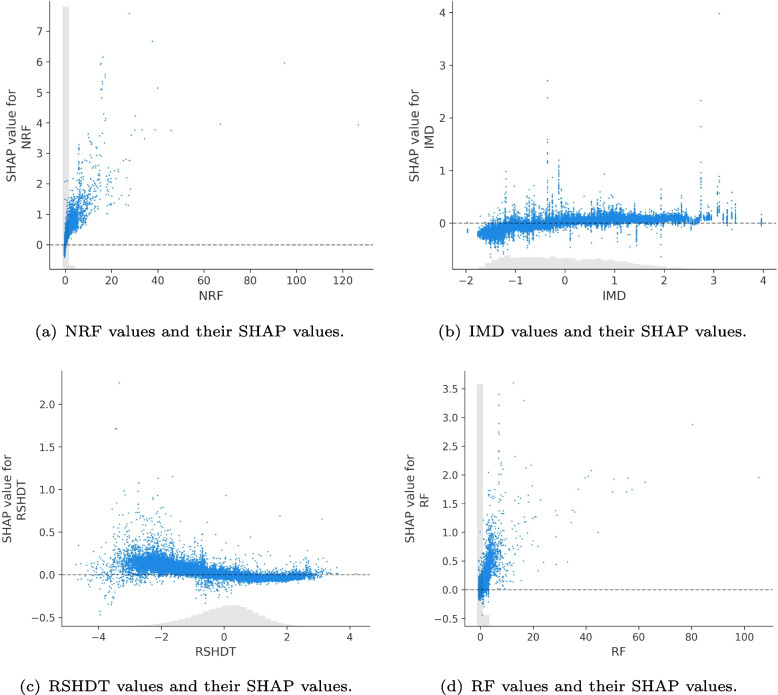
The dependency plot of the top four explanatory variable values (standardised) and corresponding SHAP values at full range

**Fig. 21:**
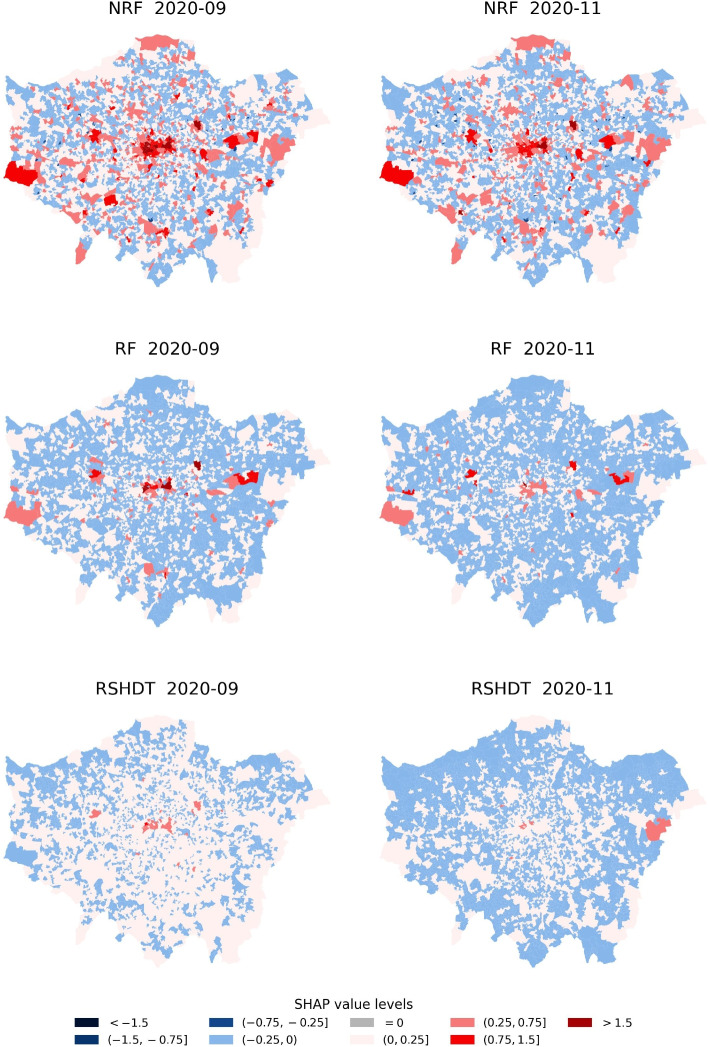
The distribution of the SHAP values of three selected variables (NRF, RSHDT and RF) during the ‘Lockdown easing period’ (represented by September 2020) and ‘Second national lockdown’ (represented by November 2020) periods

To examine the shifting of the local impact of all mobility variables and IMD on burglary levels in single LSOAs during the different observation periods, further analysis selected four LSOAs as an example (see Fig. 12) to explore the shifting in the impacts of various explanatory variables on burglary levels during two distinct pandemic periods. Figure 13 illustrates the force plotting of SHAP values for several variables (with standardised values) in February 2020 (‘Before lockdown’) and April 2020 (‘First national lockdown’), respectively. The length of the vertical bar represents the magnitude (measured by the SHAP absolute value) of the contribution/impact of each variable on burglary level prediction. A longer bar indicates a stronger impact (higher absolute SHAP value) and vice versa. The colour indicates the direction of the variable/feature’s impact on the prediction of burglary levels: red shows a positive (increased) impact while blue shows a negative (decreased) impact on the burglary level prediction. The standardised values of each variable are labelled under their respective vertical bars.

In the sub-figure in Fig. 13 corresponding to LSOA A (LSOA index in E01000599) in February 2020 (before the lockdown), the IMD (with a standardised value of 2.74) exhibited the largest and positive SHAP value in comparison to other variables. In the first national lockdown (e.g., April 2020), NRF standardised values in LSOA A decreased to −0.32 and had the largest and negative SHAP value (i.e., the longest bar in blue denoting a high and negative SHAP value of NRF). Accordingly, the standardised values of burglary level (i.e., the *f*(*x*) predicted by the XGBoost model) in LSOA A have been shifted from 0.27 (the observed value of burglary level is 2.95 and the observed number of burglary incidents is 5) in February 2020 to −0.32 (the observed standardised value of burglary level is −0.73 and observed burglary number is 0) in April 2020. This means that the model predicts lower crime levels in lockdown because of the lower mobility variable values. Another shifting pattern can be seen in the sub-figure of LSOA D (LSOA index in E01001885): the SHAP values of IMD consistently exhibit a high level and stay positive (above 0) in the selected two months. There is an observable increase (i.e., the length of the blue area became longer) in the absolute SHAP values of NRF (with the NRF value reduced from −0.19 to −0.32) from ‘Before lockdown’ to ‘First national lockdown’ in LSOA D. In addition, a noticeable transition is also observed wherein residents’ stay-at-home duration time (RSHDT) increased from −1.16 in February 2020 to 0.63 in April 2020 (as restriction policy during the first national lockdown), while the SHAP value of RSHDT changed from a positive value in February 2020 to a negative value in April 2020.

Considering the shifting of SHAP values of features in LSOA B (LSOA index is E01004091) and LSOA C (LSOA index is E01000707), it is observed that the low-level IMD value (−1.55 for LSOA B and −1.67 for LSOA C) obtained significant negative SHAP values (below zero) in February and April 2020. The SHAP values of population mobility variables (e.g., NRF, RRG and RMDH) shifted from positive to negative (the impact of these mobility variables on the burglary levels switched directions) from normal month to restriction month. The shifting of SHAP values for certain variables is also evident in LSOA C, which is concomitant with alterations in the variable’s value levels across distinct pandemic periods (from ‘Before lockdown’ to ‘First national lockdown’).

## Discussion

The present study sought to understand how burglary incident levels are impacted by the collective mobility (movements and visiting) of residents and non-residents inferred from geo big data throughout London’s neighbourhood areas and focusing on distinct social change scenarios during the pandemic periods. The analytical methodologies have demonstrated that the movement and visiting variables of residents and non-residents can be efficiently measured and observed to be associated with burglary levels. In the analytical approach, residents and non-residents in local LSOAs were first distinguished based on mobility trajectory patterns. Subsequently, movement and visiting variables were quantified for residents and non-residents at LSOA- and month-levels. An explainable machine learning approach incorporating the XGBoost regression model and SHAP strategy was used to deconstruct the impact of mobility and neighbourhood disadvantage variables on the burglary levels in various London LSOAs over the two-year observational period. Relying on SHAP values as an impact metric, this study has addressed the three research questions posed in the Introduction by providing empirical evidence on the relationship between collective mobility and burglary in urban neighbourhoods.

First, the study found that the collective mobility patterns of residents and non-residents differentially influence burglary levels in local neighbourhoods. The global results reveal that higher non-resident footfall is associated with increased burglary levels, while the extended residents’ stay-at-home duration time is correlated with reduced burglary levels in urban neighbourhood areas. Second, this study also found that the influence of mobility on burglary levels varies across neighbourhoods with different levels of deprivation. Local examination of specific local urban neighbourhood areas (LSOAs) showed the influences of mobility variables on burglary levels were moderated by the neighbourhood’s deprivation levels. Third, the results show that both global and local impacts of collective mobility varied across different societal conditions. In particular, local impact analysis indicated that residents’ footfall and time spent at home influence burglary differently depending on neighbourhood deprivation levels. Collective mobility effects on crime were also moderated by pandemic-related restrictions and easing measures. Overall, this investigation demonstrated that there were a varied set of mechanisms through which population collective mobility influenced burglary levels across different local neighbourhoods and social conditions.

The observed heterogeneity in the results demonstrates that the interactions between movement and visiting variables and levels of neighbourhood deprivation differentially influence burglary levels. This indicates that complex mechanisms are in play and understanding neighbourhood burglary levels from a singular perspective would fail to account for the specific mechanisms of crime within neighbourhoods driven by human mobility patterns. For instance, while higher neighbourhood deprivation is generally associated with increased burglary incident levels as been found elsewhere (Tilley et al. 2011), such risks also interact with other population mobility factors.

Considering the shifting impacts of collective mobility and neighbourhood deprivation on burglary levels across diverse observation periods and specific urban neighbourhoods, it is observed that the influence of population-based opportunity manifests a pronounced dynamic and changing pattern. One potential explanation is that pandemic-induced lockdowns significantly changed natural surveillance mechanisms, thereby influencing offender target selection in urban neighbourhoods. In normal times, the high volumes of non-resident footfall traffic can diminish the efficacy of natural surveillance, thereby potentially reducing the perceived risk of potential burglars being identified or reported by local residents in neighbourhoods. During lockdown, the restriction policy disrupted the population activities (like travelling to other neighbourhoods) so as to potentially changing the burglary risks in urban areas. Local residents’ mobility was also restricted during the restriction period, as demonstrated by the extended stay-at-home duration time and reduced travel distance, also contributing to the local guardianship of local neighbourhoods against burglary crimes.

A further explanation is that mobility (movement and visiting behaviour) patterns are associated with the socioeconomic conditions of local residents, which in turn shape the dynamic opportunities of burglary in neighbourhoods. Specifically, the socio-economic status of residents critically determines their mobility patterns, which reflects the range of opportunities available in their immediate surroundings (Chen et al. 2023). Furthermore, the offender population mobility patterns are also related to area-level and individual socio-economic conditions which affect the decision-making process. This process involves identifying general urban areas deemed to be more conducive to offending and then choosing particular locations to commit crimes (Clarke 1995; Brantingham 2016; Bernasco and Block 2009).

Analysing the collective mobility of both non-residents and local residents in each neighbourhood is crucial for understanding the crime generation mechanisms. It not only assists in pinpointing high-risk patterns related to the dynamic population’s mobility but also provides insight into the types of neighbourhoods where specific mobility patterns have a pronounced impact on crime occurrence. For example, a neighbourhood characterised by low deprivation that attracts non-resident visitations might exhibit distinct burglary risks compared to an area with a higher level of deprivation. Hence, it is vital to recognise how different population groups and their mobility behaviours influence burglary within urban neighbourhoods, especially during changing social contexts. With a comprehensive understanding of mobility patterns and neighbourhood characteristics, authorities and policymakers can develop tailored strategies to combat neighbourhood burglary effectively. For instance, in neighbourhood areas where burglary spikes correlate with increased non-resident visitation, strategies like heightened police patrols during peak hours or enhanced home security measures have promise in being effectively deployed.

There are several limitations of the analysis that should be articulated concerning this study. First, the effectiveness of classifying the population (from mobile phone users) to residents or non-residents using the method in this study remains unverified. The heuristic method of home location detection applied in this study may not adequately consider the individual mobility complexities leading to potential inaccuracies in the classification of different population groups. For example, hotels and other temporary accommodations can introduce bias as residential short-term stays in such locations will not accurately represent an individual’s true home location. To address this, one approach would be to apply alternative methods that leverage location records across different time spans. For example, there might be a frequency of location rule that defines a stay as being taken by a ‘resident’ and excludes the stays within hotel or accommodation locations. This helps to infer the home location more reliably and in turn allows for more precise classification of resident and non-resident populations. This method would however have the practical drawback of involving many complex calculations that track individuals in the data over time. Furthermore, a more precise classification of population groups could be considered as a potential direction for further analysis. For example, distinguishing between the movements of employed and unemployed populations. This would better capture the varying impacts of different population activities on crime patterns during weekly cycles and periods of social change. Second, the current analysis focuses solely and directly on the interplay between guardianship as measured by collective mobility and the impact of social conditions on burglary levels, without examining the influence of different built environments and urban land use within neighbourhoods. Additionally, the extent of the impact of collective mobility factors on burglary levels within specific geospatial units requires further investigation which might suggest a differently sized unit of analysis is appropriate. Since burglars often operate across nearby neighbourhood areas which can leads to the near-repeat patterns in burglary (Bowers and Johnson 2005; Chen et al. 2020), the impacts of crime and mobility in urban areas may spill over into adjacent areas. Therefore, explicitly incorporating spatial and temporal dependencies as input variables could enhance model performance in future analyses. Third, the impact of diversity of mobility behaviours (measured as the resident’s mobility entropy in this study) on crime levels in neighbourhoods requires further exploration as evidence for a significant impact was not found in this study. In parallel, the duration of stay for residents and visitors could also be further explored in urban areas as stay-at-home duration has been found to have a significant association with crime levels in this study. For example, it might be that the degree to which high footfall is criminogenic or protective at place varies with the typical length of stay in those locations. Fourth, this study does not undertake causal inference analysis but can only examine associations in terms of impact and therefore experimental approaches are required to provide stronger evidence in terms of policy implications. This study is therefore limited in its ability to draw clear causal connections between the collective mobility and burglary levels examined in different contexts of social conditions.

## Conclusions

This study investigated the impact of collective mobility of local and non-local residents on burglary levels through the utilisation of explainable machine learning techniques in London’s neighbourhood areas from 2020 to 2021. The analysis revealed that collective mobility sensed from geo big data showed a strong impact on burglary levels, especially in terms of the frequency of non-residents’ visitations and the time duration of residents’ stays at home. This interplay between mobility, neighbourhood deprivation and crime changes demonstrates various contextual mechanisms of burglary shifting across local areas. This analysis has also underscored the relevance of considering local population mobility patterns in formulating more precise crime mitigation measures. In further studies, an in-depth and high-resolution spatial-temporal analysis of local residents’ mobility patterns with other crime types may offer a deeper understanding of their interactions.

## Data Availability

The data and materials in this work are available upon request.

## Footnotes

1 Office for National Statistics: https://www.ons.gov.uk/

2 Data.police.uk: https://data.police.uk/

3 Sources: Location Sciences AI, now known as Sorted: https://sorted.com/

4 Ministry of Housing, Communities & Local Government: https://www.gov.uk/government/statistics/english-indices-of-deprivation-2019

5 GridSearchCV: https://scikit-learn.org/stable/modules/generated/sklearn.model_selection.GridSearchCV.html
